# Devising Mixed-Ligand Based Robust Cd(II)-Framework From Bi-Functional Ligand for Fast Responsive Luminescent Detection of Fe^3+^ and Cr(VI) Oxo-Anions in Water With High Selectivity and Recyclability

**DOI:** 10.3389/fchem.2021.651866

**Published:** 2021-05-05

**Authors:** Manpreet Singh, Gaurav Kumar, Subhadip Neogi

**Affiliations:** ^1^ Inorganic Materials and Catalysis Division, CSIR-Central Salt and Marine Chemicals Research Institute (CSMCRI), Bhavnagar, India; ^2^ Academy of Scientific and Innovative Research (AcSIR), Ghaziabad, India

**Keywords:** metal-organic frameworks (MOFs), hydrolytic stability, luminescent sensing, water contaminants, fast responsive detection

## Abstract

Environmental issue related applications have globally surfaced as hottest areas of research, wherein luminescent metal-organic frameworks (LMOFs) with functionalized pores put unique signature in real-time monitoring of multiple classes of toxic compounds, and overcome many of the challenges of conventional materials. We report a two-fold interpenetrated, mixed-ligand Cd(II)-organic framework (**CSMCRI-11**) [Cd_1.5_(**L**)_2_(*bpy*)(NO_3_)]·DMF·2H_2_O (CSMCRI = Central Salt and Marine Chemical Research Institute, H**L** = 4- (1H-imidazol-1-yl)benzoic acid, *bpy* = 4,4′-bipyridine) that exemplifies bipillar-layer structure with two different Cd(II) nodes, and displays notable robustness in diverse organic solvents and water. Intense luminescence signature of the activated MOF (**11a**) is harnessed in extremely selective and fast responsive sensing of Fe^3+^ ions in aqueous phase with notable quenching constant (1.91 × 10^4^ M^−1^) and impressive 166 ppb limit of detection (LOD). The framework further serves as a highly discriminative and quick responsive scaffold for turn-off detection of two noxious oxo-anions (Cr_2_O_7_^2−^ and CrO_4_^2−^) in water, where individual quenching constants (CrO_4_^2−^: 1.46 × 10^4^ M^−1^; Cr_2_O_7_^2−^: 2.18 × 10^4^ M^−1^) and LOD values (CrO_4_^2−^: 179 ppb; Cr_2_O_7_^2−^: 114 ppb) rank among best sensory MOFs for aqueous phase detection of Cr(VI) species. It is imperative to stress the outstanding reusability of the MOF towards detection of all these aqueous pollutants, besides their vivid monitoring by colorimetric changes under UV-light. Mechanism of selective quenching is comprehensively investigated in light of absorption of the excitation/emission energy of the host framework by individual studied analyte.

## Introduction

Severe pollution of earth’s hydrosphere by detrimental chemicals has raised global concerns to human health and ecological systems. (Samanta et al., 2019; Yao et al., 2020) For instance, Fe^3+^ ion is one of the vital ions existing in the living organism and plays essential role in transportation of oxygen molecule through blood. It also has a fundamental role in the formation of haemoglobin, enzyme and proteins. (Xu et al., 2016) Hence, Fe^3+^ ion is firmly an indicator of health. Both inadequacy and abundance in blood plasma can lead to biological/genetic malfunction. Syndrome like insomnia, cancer, heart and declining immunity is correlated with iron content in the human body. (Dang et al., 2012; Liu et al., 2016; Chandra Rao and Mandal, 2018) Also, in the physiological milieu, iron exists as Fe^2+^ and Fe^3+^ are essentially playing a significant role So, it is imperative to detect one from the other because a specific amount of Fe^3+^ is needed to promote the formation of muscle and haemoglobin. (Barba-Bon et al., 2012; Gogoi and Biswas, 2018) Though a wide range of literature is available for sensing of Fe^3+^ ions but sensing under aqueous condition is still rare. (Xu et al., 2016; Yang et al., 2019) In this direction, the readiness of new sensors for Fe(III) ion detection is as yet a challenging objective. (Lustig et al., 2017; Sun et al., 2019) Alongside, chromium is also essential for mankind. Cr (III) is an essential biological element while Cr(VI) ion as CrO_4_^2−^ and Cr_2_O_7_^2−^ oxo-anions is employed as an oxidising agent in manufacturing processes (Lv et al., 2016). Owing to exhaustive use in industrial processes, these oxo-anions are utterly polluting the environment and water bodies as a result of their outstanding solubility in water. These two oxo-anions are both cytotoxic and carcinogenic, and leads to the disruption of various proteins, enzymes and DNAs in the animal body. (Dong et al., 2016; Chen et al., 2017a; Dong et al., 2017; Sun et al., 2019) Thus, it is of vast significance to monitor minute presence of Fe^3+^ and/or Cr(VI) ions in aqueous media via highly sensitive, fast-responsive and easily portable method. (Xu and Yan, 2015; Li et al., 2019; Singh et al., 2020)

**SCHEME 1:**
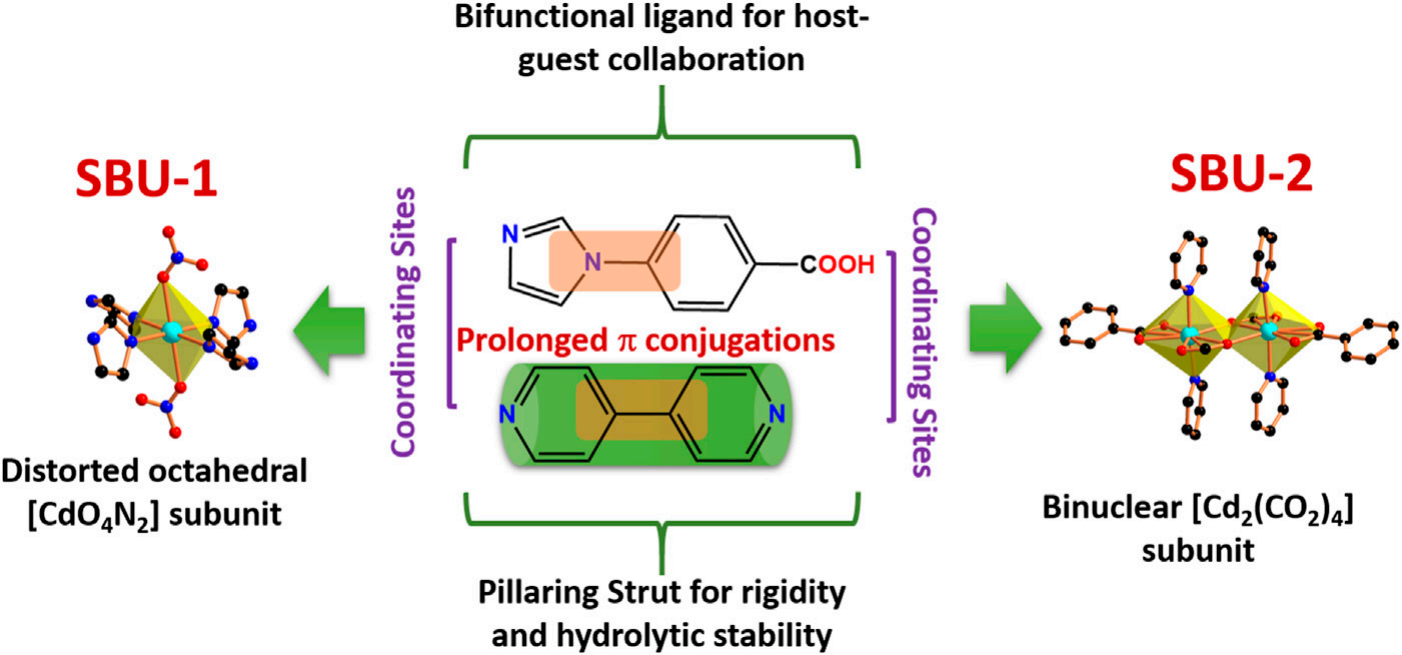
Notable Features of the H**L** (bifunctional ligand), *bpy* (pillaring strut) and Cd^2+^ Nodes/Units in **CSMCRI-11.**

As of now, conventional procedures include high-performance liquid chromatography (HPLC), ion-exchange chromatography, gas chromatography (GC), gas chromatography-mass Spectrometry (GC/MS), ion-exchange chromatography and other detection techniques. Those techniques are time-consuming, quite expensive and require complicated instruments. On the other hand, luminescence-based sensory materials have shown several advantages, for instance, fast response time, ease of performance, distinct signal outputs, low cost, high sensitivity, and low detection limit. (El Rassi, 1997; Zhu et al., 2013; Wang et al., 2015; Senthilkumar et al., 2018; Guo et al., 2019; Yao et al., 2020) Although, several homogenous sensors have been developed, (Ma et al., 2019), quest of heterogeneous sensor is indispensable owing to their advantages like separation, eco-friendly nature and multicyclic regeneration properties. (Liu et al., 2016; Vikrant et al., 2018; Zhao et al., 2018; Yan, 2019) In this milieu, metal-organic frameworks (MOFs) have grown as most fascinating porous, heterogonous scaffolds with well defined-crystalline materials, and recently used as probes for luminescent sensing of diverse lethal pollutants. (Kitagawa et al., 2004; Chen et al., 2007; Cohen, 2010; Cui et al., 2011; Kreno et al., 2012; Zhou et al., 2012; Pettinari et al., 2017; Zhang et al., 2017) In relation to the aforesaid concerns, coupled with our ongoing efforts to develop new sensory LMOFs for minute detection of diverse lethal pollutants, (Goswami et al., 2019a; Goswami et al., 2019b; Singh et al., 2020; Seal et al., 2021a; Seal et al., 2021b) we have synthesised a water stable, Cd(II)-MOF **CSMCRI-11** using the bifunctional ligand 4- (1H-imidazol-1-yl) benzoic acid (H**L**, Supplementary Scheme S1) in combination with pillaring linker *bpy* (Scheme 1). **CSMCRI-11** exhibits two-fold interpenetrated structure with unidirectional pores. This framework shows highly selective and sensitive detection of Fe^3+^ ion and noxious Cr(VI) oxo-anions (CrO_4_^2−^ and Cr_2_O_7_^2−^) at ppb level in the aqueous phase. Fast responsive time and excellent sensitivity along with very low limit of detection render this framework one of the best LMOFs among contemporary reports, and promises its utilization as smart material for detection of hazardous contaminants in the aqueous phase.

## Results and Discussion

### Crystal Structure of CSMCRI-11

Framework **CSMCRI-11** was synthesized as colourless rectangular crystals under solvothermal conditions by reaction of bifunctional ligand 4- (1H-imidazol-1-yl) benzoic acid (H**L**), 4,4′-bipyridine (*bpy*) and Cd(NO_3_)_2_·4H_2_O in the molar ratio of 0.2:0.2:0.16. As-synthesized **CSMCRI-11** crystal was examined by single-crystal X-ray diffraction (Supplementary Table S1) which displays monoclinic space group *C2/c*. The asymmetric unit contains two Cd(II) ion, one *bpy* linker, two deprotonated ligand (**L** ^**−1**^: hereafter ***L***) and one nitrate anion. Both the metal centres exhibit different coordination environments. For instance, Cd(1) is coordinated with four oxygen atoms from the carboxylate group of ***L*** and two nitrogen atoms (N atoms N3 and N5) from the pyridyl ring of *bpy* linker in distorted octahedral geometry (CdO_4_N_2_). The Cd-O bond length range from 2.292 to 2.419 Å while Cd-N bond length ranges from 2.285 to 2.297 Å. (Zhai et al., 2019).

Two alike Cd (1) ions are bridged through two carboxylate groups in *syn*-*anti* fashion to form a binuclear [Cd_2_(CO_2_)_4_] subunit (Scheme 1) with Cd(1)-Cd(1) spacing of 3.986 Å. On the other hand, Cd (2) atom is in entirely different coordination milieu, and reveals distorted octahedral geometry (CdO_4_N_2_) via ligation with four imidazolyl nitrogen atoms of ***L*** in the equatorial plane, and two oxygen atoms of nitrate anions. Here, Cd-O bond length is 2.358 Å, and Cd-N bond length varies from 2.296 to 2.318 Å. Every deprotonated ligand is in coordination with [Cd_2_(CO_2_)_4_] subunits and Cd (2) ions forming two-dimensional (2D) layer along the crystallographic *b* axis.

**FIGURE 1:**
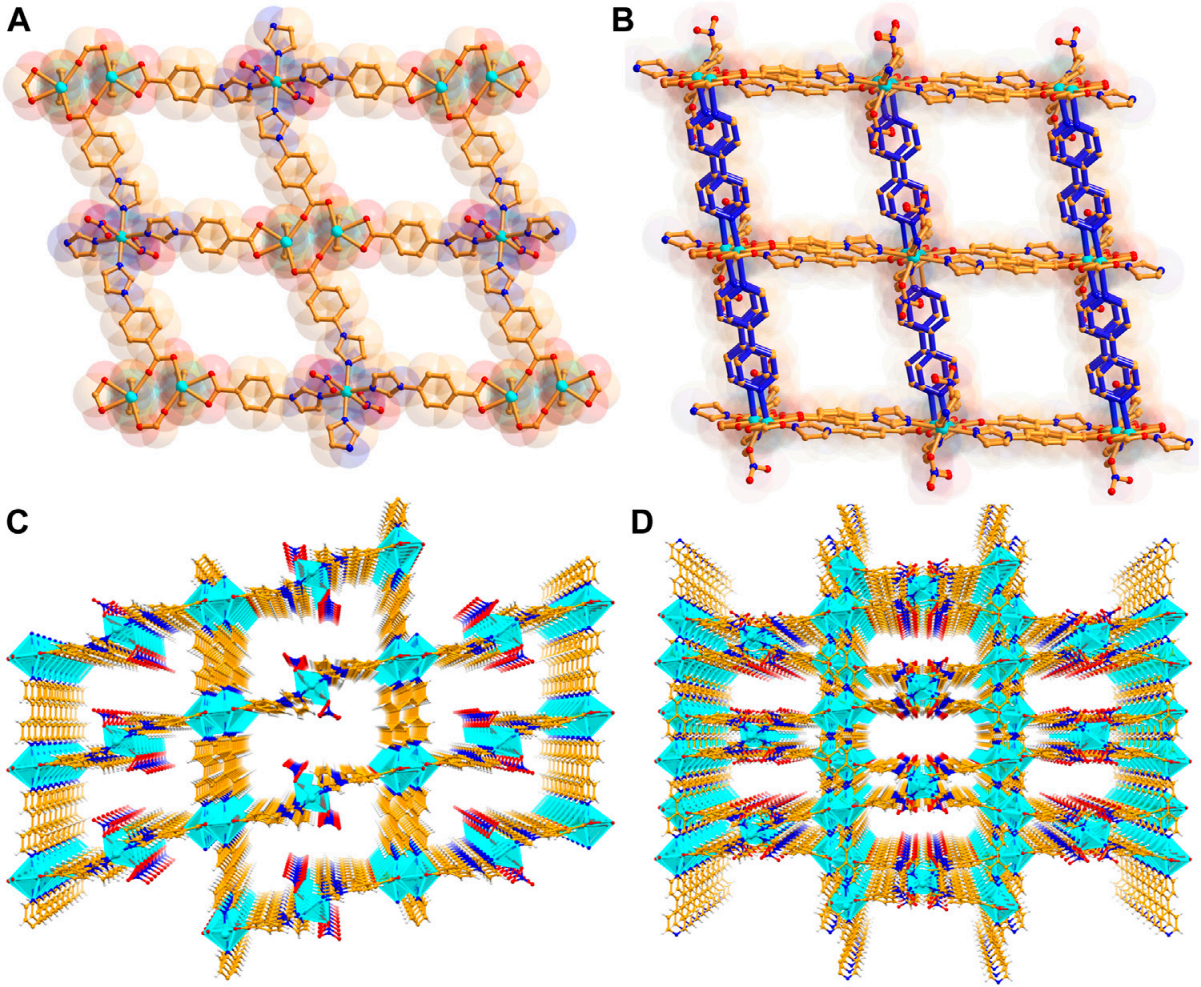
**(A)** A view of the 2D layer structure along *ac* plane, and **(B)** simplified demonstration of bipillar-layer structure, **(C)** tilted view of the one-dimensional porous channel in non-interpenetrated and **(D)** interpenetrated structure along *c* axis.

The weavy layer acts as a roof with rhombus window (Figure 1A) of size 16.4 × 18.8 Å^2^ (considering atom to atom connection). Two *bpy* linkers connects every [Cd_2_(CO_2_)_4_] units, leading to a double-pillar layered three-dimensional (3D) structure with the formation of large voids (dimension: 11.4 × 24.5 Å^2^) along the *c* axis. Such huge pores mutually instigate two-fold interpenetration (Figure 1D) to the overall structure of **CSMCRI-11**. Nevertheless, rectangular cavities of dimension 6.54 × 18.09 Å^2^ still exists along ***ab*** plane. Also, pillaring *bpy* linkers are accompanied by string π–π interaction (centroid–centroid distances in the range: 3.794 Å), imparting high rigidity to the 3D porous network.

These porous channels accommodate DMF and H_2_O solvent molecules, which are highly disordered. However, their presences were alternatively ascribed from a combination of PLATON calculation, thermogravimetric weight loss, elemental analysis, and IR spectral data that corresponds to the molecular formula of **CSMCRI-11** [Cd_1.5_(L)_2_(*bpy*)(NO_3_)]·DMF·2H_2_O. Regardless of two-fold interpenetration, the solvent accessible voids of **CSMCRI-11** is estimated to be 31.6% of the total crystal volume.

### Assessment of Thermal and Moisture Stability of CSMCRI-11

**FIGURE 2:**
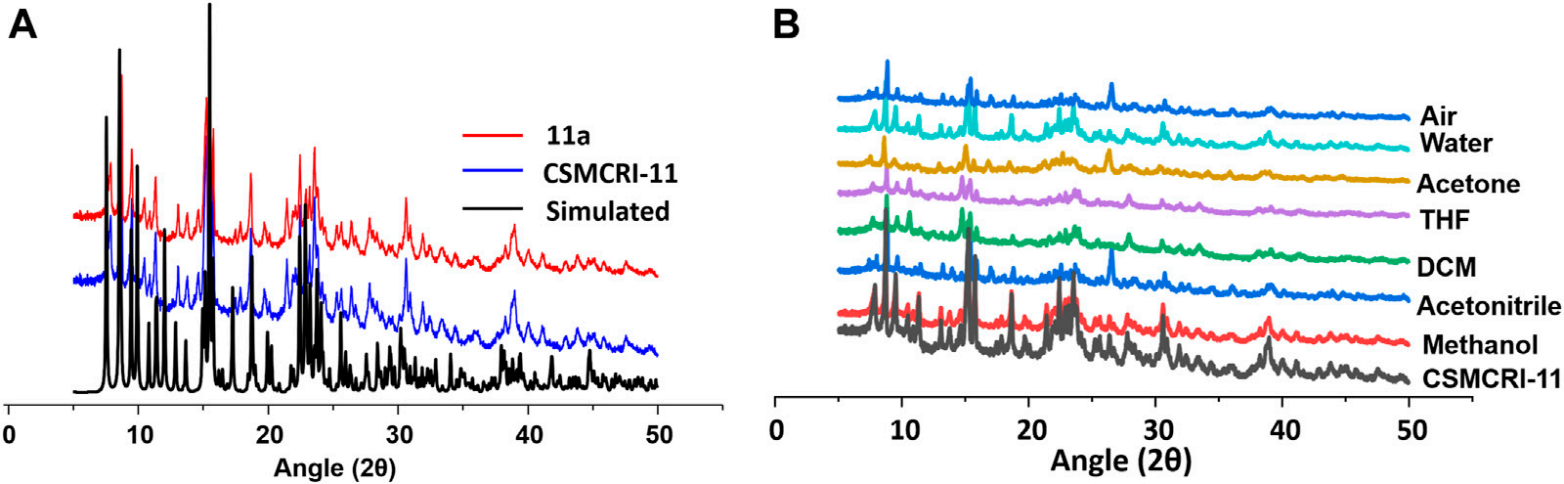
**(A)** PXRD pattern of **CSMCRI-11** calculated, synthesised and activated framework. **(B)** PXRD curves of the pristine framework **CSMCRI-11** in diverse solvents and air.

The purity of bulk phase and structural integrity of **CSMCRI-11** was established from strong correlations of peaks between powder X-ray diffraction (PXRD) pattern of as synthesised framework and simulated one (Figure 2A), obtained from crystallographic data. The FT-IR spectral data exhibits IR band of the >C═O (carbonyl) for guest DMF solvent at 1663 cm^−1^ (Supplementary Figure S4). Also, a broad absorption peak centred around 3300 cm^−1^ corresponds to lattice water molecules. Thermal stability of the framework was assessed from thermogravimetric analysis (TGA) under inert atmosphere (Supplementary Figure S5), showing a two-step weight-loss. The first weight loss was observed in the temperature range 30–100 ^o^C and corresponded to 3.89% of the initial mass, accounting for the removal of lattice water. The second weight loss of 8.89% features loss of DMF solvents. A sharp weight loss beyond 350 ^o^C relates to framework decomposition. For, generation of the guest-free (activated) framework (hereafter **11a**), the as-synthesised crystals of **CSMCRI-11** were immersed in methanol for three days (by exchanging the solvent three times a day), and solvent exchanged framework was heated under vacuum at 120 ^°^C for overnight. The FT-IR spectra analysis of **11a** shows absence of all the peaks related to H_2_O or DMF solvents. Furthermore, the TGA curve of **11a** did not reveal any weight loss up to 320 ^°^C, supporting its robust nature at high temperature (Supplementary Figure S5). To further corroborate stability of the structure, 50 mg of as-made **CSMCRI-11** was taken in a vial and exposed to common organic solvents (methanol, acetonitrile, dichloromethane, tetrahydrofuran, acetone), and water. Importantly, PXRD patterns of these exposed samples remained unchanged (Figure 2B). We further investigated the hydrolytic stability of the framework by exposing it to saturated water vapours at room temperature. A time-dependent PXRD (Supplementary Figure S6) study up to 10 days was carried out at a regular interval, which revealed maintenance of structural integrity throughout, and substantiates to sufficient hydrolytic stability of the MOF. The stability aspect was further assessed by leaving **11a** in the open air for 15 days. Remarkably, PXRD pattern (Figure 2B) remained unchanged and corroborates to its high robustness.

### Photoluminescence Studies

Strategic incorporation of electronically passive transition metal in association with conjugated organic struts is one of the potential strategies for the synthesis of luminescent MOFs. (Zhu et al., 2015; Zhang et al., 2018; Zhou et al., 2018) Owing to the excellent thermal and hydrolytic stability, together with its highest luminescence intensity in water (Supplementary Figure S7c), **CSMCRI-11** was employed as a fluorescent probe in activated form for aqueous phase minute detection of water contaminants. Upon excitation at 265 nm, aqueous dispersion of **11a** showed strong emission band at 426 nm that is attributed to mixed contribution of the intra-ligand charge transfer of π-electron-rich links, (Chen et al., 2007) ligand-to-ligand charge transfer (LLCT) (π−π* and n−π* transitions) (Luo et al., 2012; Wan et al., 2015) and adequate π−π stacking interaction between the ligands in the structure. (Zhao et al., 2016; Liu et al., 2019) Additional experiment showed that after filtration of MOF particles, no fluorescence (Supplementary Figure S7d) could be observed in the remaining solution supporting the fact that above emission originates as a result of dispersed MOF particles only. UV-visible spectrum (Supplementary Figure S7a) of **11a** shows a redshift of 9 and 3 nm in comparison to *bpy* and H**L**, respectively that are associated with binding of linkers to the Cd(II) metal ions. Remarkably, the intensity of the photoluminescent (PL) spectrum of **11a** was found to be > 90% higher than constituting ligands. Coordination interactions through Cd(II) centres enhance the rigidity of the structure and boosts the PL intensity to such extent.

### Luminescent Sensing of Fe^3+^ Ions in ppb Level

**FIGURE 3:**
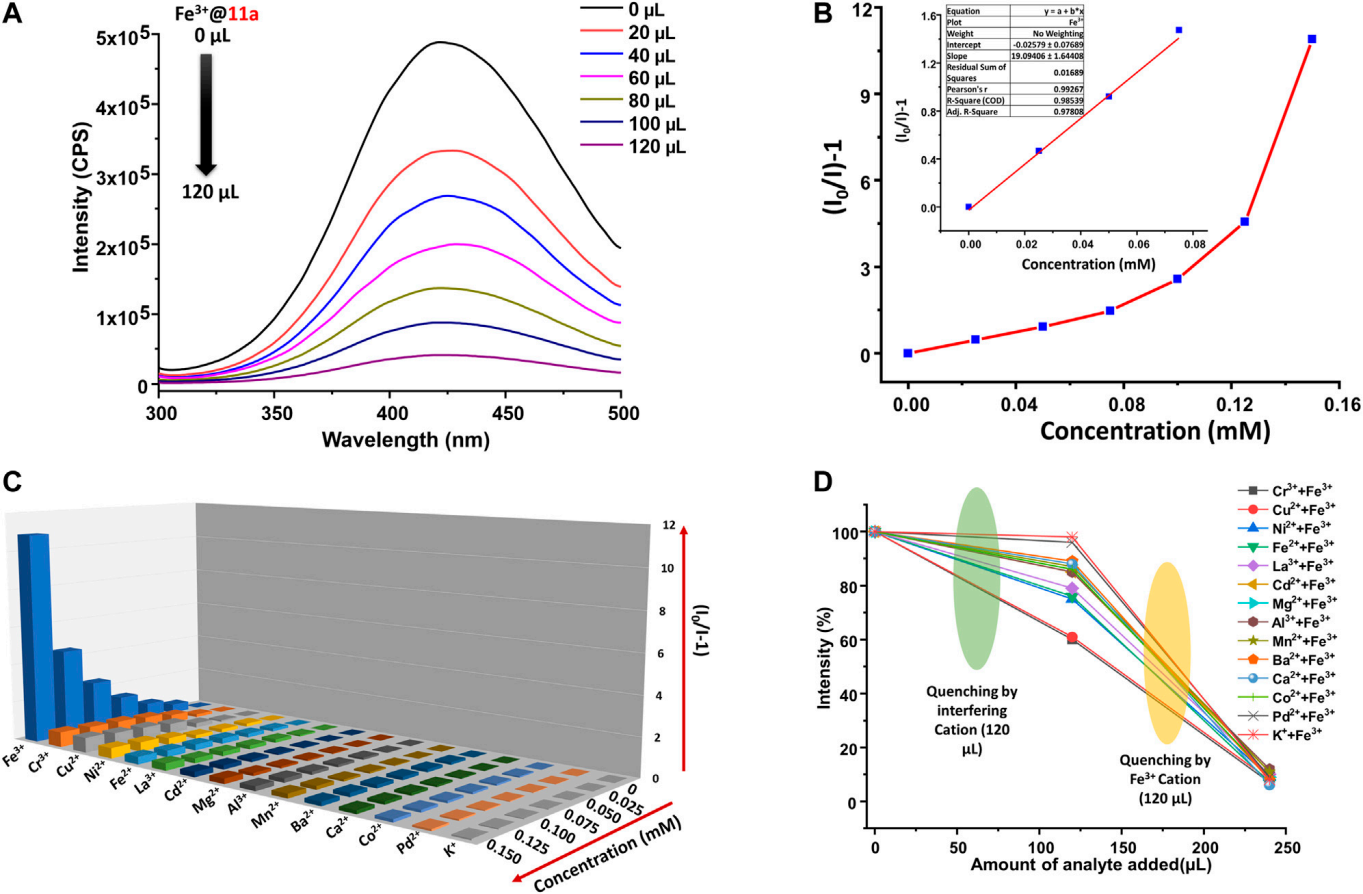
**(A)** Photoluminescence spectra of **11a** upon gradual addition of aqueous solution Fe^3+^ ions (2.5 mM), **(B)** Stern-Volmer plot for **11a** upon incremental addition of Fe^3+^ ions (Inset: linear region of the plot), **(C)** S-V plots for all studied metal ions (2.5 mM) upon titration with well-dispersed suspension of **11a** (λ_max_ of emission **11a** ca. 426 nm; 5 nm slit width), **(D)** Interference plot presenting the decrease in PL intensities upon addition of aqueous solution of several metal ions (2.5 mM, 120 μl), followed by Fe^3+^ ions (2.5 mM, 120 μl).

Owing to high photoluminescence intensity, **11a** has been employed as a probe for selective and sensitive detection of metal ions in the aqueous phase. 1 mg of powdered **11a** was dispersed in 2 ml water and sonicated for 2 h to generate a uniform suspension (1 mg/2 ml dispersion of **11a** for each titration) Photoluminescence spectra of the aqueous phase dispersion was recorded by incremental addition of a series of MCl_y_ metal salts (M: Ba^2+^, Fe^3+^, Fe^2+^, K^+^, Mn^2+^, Ca^2+^, Cd^2+^, Pd^2+^, Co^2+^, Al^3+^, Ni^2+^, Cu^2+^, Cr^3+^, Mg^2+^, and La^3+^) in water (2.5 mM). For all these cases, the luminescence spectra were recorded at an emission wavelength of 426 nm under constant stirring at room temperature upon incremental addition of analyte (20–120 µl), and every titration was performed in triplicates to ensure the reproducibility of the results. Surprisingly, a rapid and significant decrease in the luminescence intensity of **11a** was detected (Figure 3A) only in case of Fe^3+^ ion solution, while remaining metal ions did not show any significant changes. The quenching efficiency of Fe^3+^ ion is found to be 91.6% (Supplementary Figure S8), whilst the rest of the metal ions showed almost nominal values. The quenching competence follows the trend (Supplementary Figure S8): Fe^3+^>Cr^3+^>Cu^2+^>Ni^2+^>Fe^2+^>La^3+^>Cd^2+^>Mg^2+^>Al^3+^>Mn^2+^>Ba^2+^>Ca^2+^>Co^2+^>Pd^2+^>K^+^.

Quantitative analysis of the fluorescence quenching of **11a** by Fe^3+^ ions was determined by Stern-Volmer (S-V) equation (Parmar et al., 2017a; Parmar et al., 2017b): I_0_/I = 1 + *K*_sv_ [C] (I and I_0_ are photoluminescence intensities of the **11a** after and before titration with analytes, respectively; [C] is the molar concentration of analytes ions in mM; *K*_sv_ is known to be S-V constant in M^−1^). Change in the PL intensity was recorded upon incremental addition of an aqueous solution of Fe^3+^ ions (2.5 mM) to the aforesaid MOF dispersion in water (Figure 3B). The bent S-V plot at higher Fe^3+^ concentration might be attributed to the dynamic and static quenching process. (Chen et al., 2018) For the calculation of *K*_SV_, a linear correlation between the PL intensity and Fe^3+^ concentration was considered at lower concentration range (0–0.08 mM), which resulted remarkable value of 1.91 × 10^4^  M^−1^ (Table 1) that stands well in literature reports for detection for Fe^3+^ ions (Supplementary Table S6). Further, limit of detection (LOD) was obtained from changes in the emission intensity on incremental addition of 10 µM solution of Fe^3+^ ions. By employing the standard equation 3σ/K (Nagarkar et al., 2014; Karmakar et al., 2017) (*σ* = standard deviation of initial intensity of the MOF without analyte for five consecutive blank measurements at 2 min intervals, K = slope of the linear curve in Supplementary Figure S9) the LOD value for Fe^3+^ turned out (Supplementary Table S3) to be 0.219 μM (corresponding to 166 ppb). Given quenching efficiency, *K*_SV_ value, and LOD are important and desired criteria for sensing application, Fe^3+^ ion sensing by **11a** truly corroborates ultra-sensitive detection in comparison to literature reports (Supplementary Table S6).

**TABLE 1:** Performance Characteristics of **11a** for Detection of Fe^3+^, CrO_4_^2−^ and Cr_2_O_7_^2−^ ions in Water.

Analyte	Fe^3+^	Cr_2_O_7_^2−^	CrO_4_^2−^
Sensing Mode	Turn-off	Turn-off	Turn-off
Quenching Extent (%)	91.60	96.13	87.15
*K*_SV_ (M^−1^)	1.91 × 10^4^	2.18 × 10^4^	1.46 × 10^4^
LOD (ppb)	166 ppb	114 ppb	179 ppb
Fast Responsive Nature	40 µl/20 s	40 µl/20 s	40 µl/20 s

To further corroborate real-time applicability, visual detection of Fe^3+^ ions was targeted in the solution phase. As divulged in Figure 7B, the high fluorescence of aqueous dispersion of **11a** under UV light (365 nm) immediately quenches upon one drop addition of Fe^3+^ solution, and authenticates to solution-phase visual detection of Fe^3+^ions.

### Selectivity, Recyclability and Fast Responsive Fe^3+^ Sensing

**FIGURE 4:**
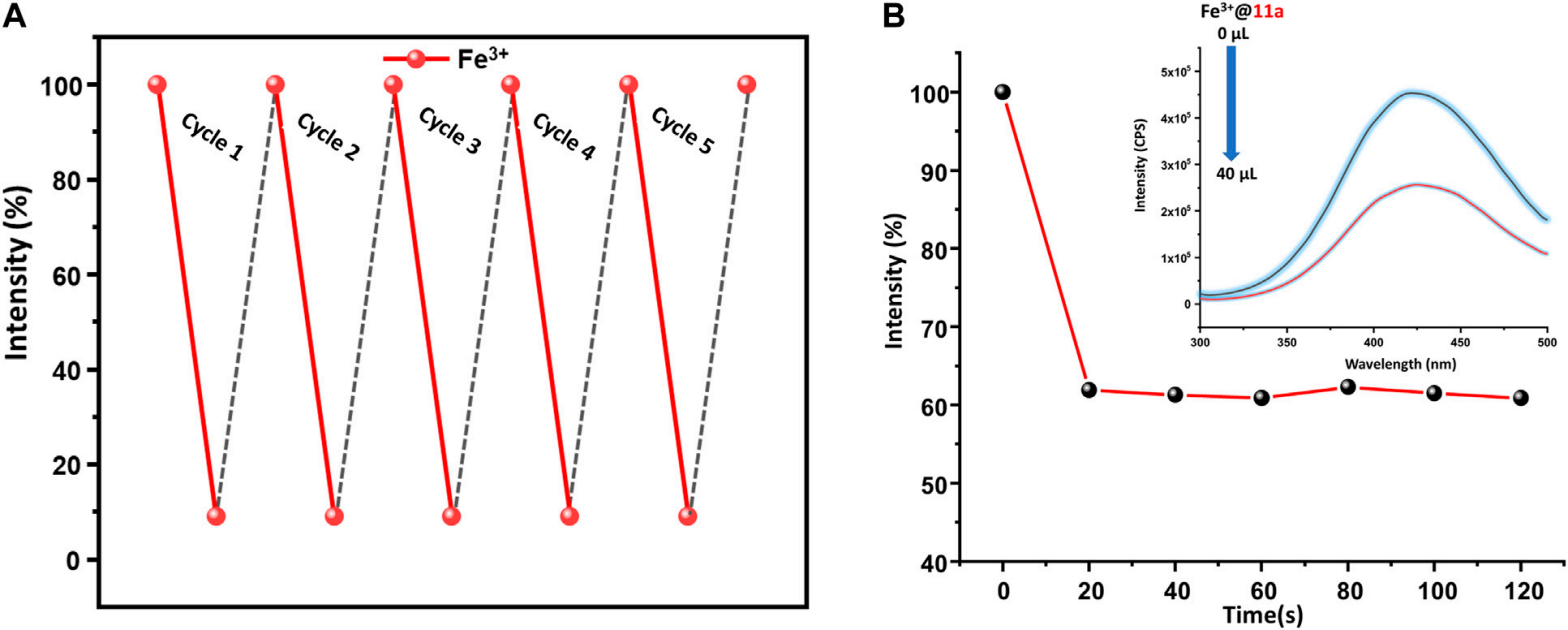
**(A)** Regeneration of pristine fluorescence intensity of **11a** towards 2.5 mM Fe^3+^ solution used for 5 cycles, **(B)** Time dependent change in fluorescence intensity of **11a**, upon addition of Fe^3+^ ion (recorded up to 120 s after a regular interval of 20 s).

Given presence of other metal ions cannot be neglected in real systems, competitive analysis test (CAT) was considered. To a 2 ml suspension of MOF, 120 µl of interfering analyte (2.5 mM) was added first, followed by similar amount of Fe^3+^ ion. While PL intensity did not change during the addition of interfering analytes, the solution exhibited more than 90% decrease of emission upon second addition. Further, to ensure the reusability of the material, MOF particles were separated via centrifugation after sensing experiments, washed thoroughly with water followed by methanol, and air-dried. The intensity of the recycled material was restored up to five consecutive sensing-recovery cycles (Figure 4A). Structural integrity and purity of reused **11a** were verified from unchanged PXRD patterns to that of pristine ones. In addition, the response time of **11a** towards sensing of Fe^3+^ was studied by recording the time-dependent change in the luminescence intensity. (Goswami et al., 2019b). For this, 40 μl of Fe^3+^ solution (2.5 mM) was added to the MOF dispersion, wherein luminescence intensity (Figure 4B) dropped to 61.9%. The change in PL intensity was recorded for 120 s after a regular interval of 20 s. Remarkably, no significant change in the photoluminescence intensity was observed beyond 20 s, and affirms fast responsive Fe^3+^ ion detection in aqueous phase.

### Luminescence Detection of Cr(VI) oxo-anions (Cr_2_O_7_^2-^/CrO_4_^2-^)

**FIGURE 5:**
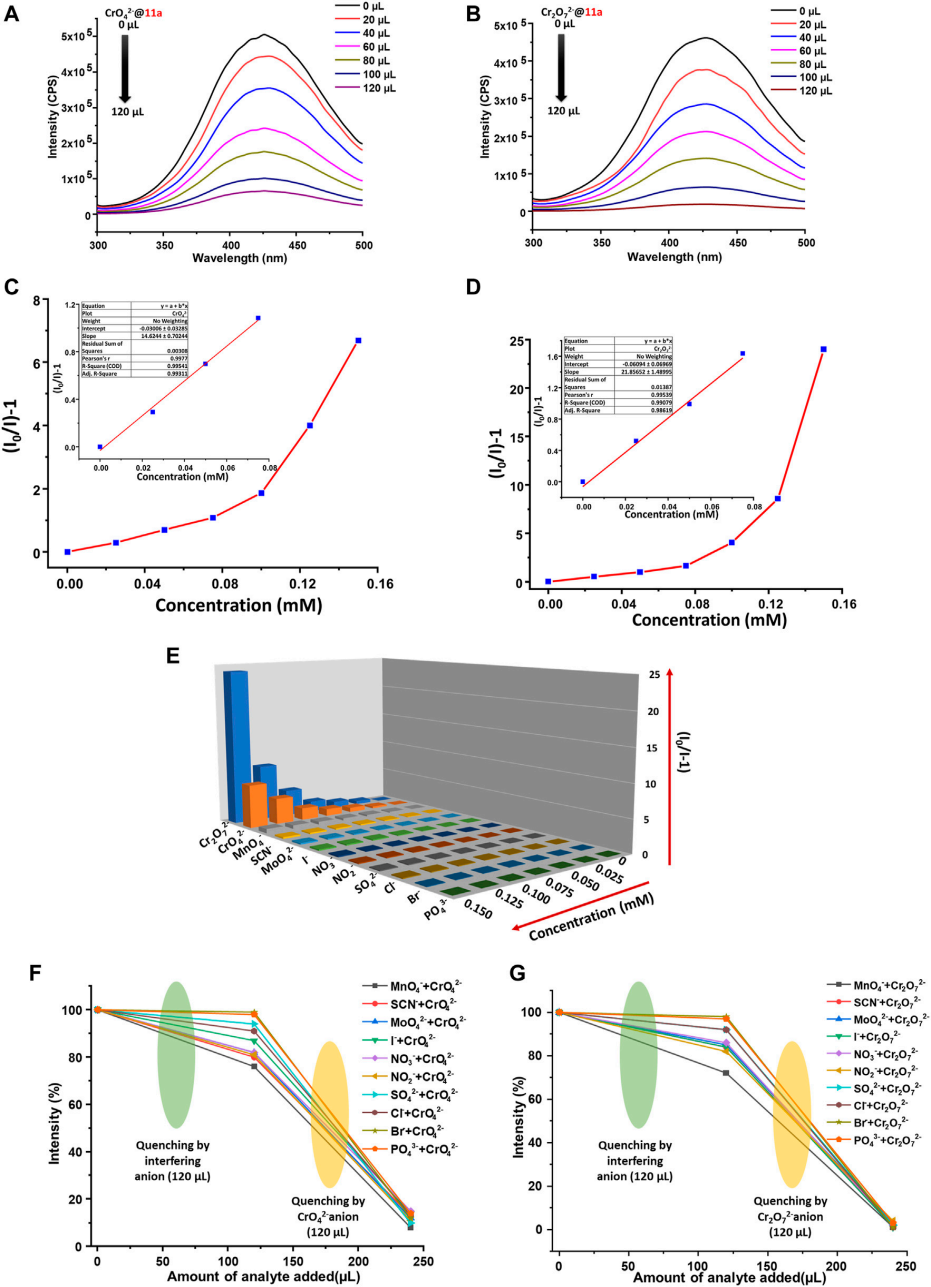
Photoluminescence spectra of **11a** upon gradual addition of aqueous solution (2.5 mM) **(A)** CrO_4_^2−^ and **(B)** Cr_2_O_7_^2−^, Stern-Volmer plot for **11a** upon incremental addition of **(C)** CrO_4_^2−^ and **(D)** Cr_2_O_7_^2−^(Inset: linear region of the plot), **(E)** S-V plots for all studied anions (2.5 mM) upon titration with well-dispersed suspension of **11a** (λ_max_ of emission **11a** ca. 423 nm; 5 nm slit width) Interference plot showing the decrease in photoluminescence intensity intensities upon the addition of aqueous solution of various anions (2.5 mM) followed by **(F)** CrO_4_^2-^ and **(G)** Cr_2_O_7_^2-^.

Selective luminescent detection of Fe^3+^ in highly sensitive and fast responsive manner inspired us to explore further application of this framework towards sensing of anions. We performed anion detection considering potassium salts of a series of anions (NO_3_^−^, SO_4_^2−^, MoO_4_^2−^, NO_2_^−^, Cl^−^, F^−^, PO_4_^3−^, SCN^−^, I^−^, MnO_4_^−^, Br^−^, CrO_4_^2−^ and Cr_2_O_7_^2−^) in water (2.5 mM). These solutions were separately added to the aqueous dispersions of **11a**. Among others, a significant quenching of PL intensity was observed by hexavalent oxo-chromium anions (Cr_2_O_7_^2−^, CrO_4_^2−^), whereas rest of the anions did not show any major changes in luminescence intensity. The order of quenching (Supplementary Figure S10) was found to be Cr_2_O_7_^2−^>CrO_4_^2−^>MnO_4_^−^>SCN^−^>MoO_4_^2−^>I^−^>NO_3_^−^>NO_2_^−^>SO_4_^2–^>Cl^−^>Br^−^>PO_4_^3−^. The quenching efficiency for Cr_2_O_7_^2−^ and CrO_4_^2−^ are 96.13 and 87.15%, respectively. For quantitative determination of the extent of quenching, changes in luminescence intensity of **11a** were recorded by incremental addition of anionic solution. Stern–Volmer constant was obtained (*vide supra*) via plotting [(I_0_/I)-1] vs. concentration of the analyte that revealed linear curve at the lower concentration (Figure 5C,D) and deviation from linearity occurs as the concentration raises. *K*_sv_ values were calculated from linear portion of the curve and found to be 1.46 × 10^4^ and 2.18 × 10^4^ M^−1^ for CrO_4_^2−^ and Cr_2_O_7_^2−^, respectively. In addition, LOD (vide supra) was evaluated by the gradual addition of 10 μM aqueous anionic solutions to 2 ml MOF dispersion. From linear fitting of the graph (Supplementary Figure S11, Supplementary Figure S12) involving intensity against analyte concentration, LOD was calculated to be 179 and 114  ppb for CrO_4_^2−^ and Cr_2_O_7_^2−^, individually. A comparison of MOF-based fluorescence sensors for the detection of lethal oxo-anionic Cr(VI) pollutants in water is tabulated in Supplementary Table S7, which indicates that quenching constants from this study rank one of the best values among existing reports. Essentially, such low detection limits render **11a** a suitable candidate for monitoring of these lethal oxo anions in water.

### Selective, Multicyclic and Fast-Responsive Detection of Cr (VI) Anions

Keeping in mind that real-system possesses many other interfering anions, we next probed selectivity of **11a** towards detection of Cr_2_O_7_^2−^ and CrO_4_^2−^ ions by performing CAT, maintaining standard protocol (*vide supra*). In a distinctive experiment, 120 µl of interfering analyte solution (2.5 mM) was added, which resulted almost insignificant changes to the emission behaviour of **11a**. Quite in contrast, successive addition of same amount of individual oxo-anionic Cr(VI) solution to the above mixture (MOF + interfering anions) leads to a drastic turn-off response to the PL intensity (Figures 5F,G). It is imperative to state that we repeated this experiment three times to ensure the chronology of results. Such remarkably selective sensing of Cr(VI) oxo-anion is of true elegance and validates **11a** as a potential and reliable probe for detection of Cr(VI) oxo-anions in aqueous phase.

**FIGURE 6:**
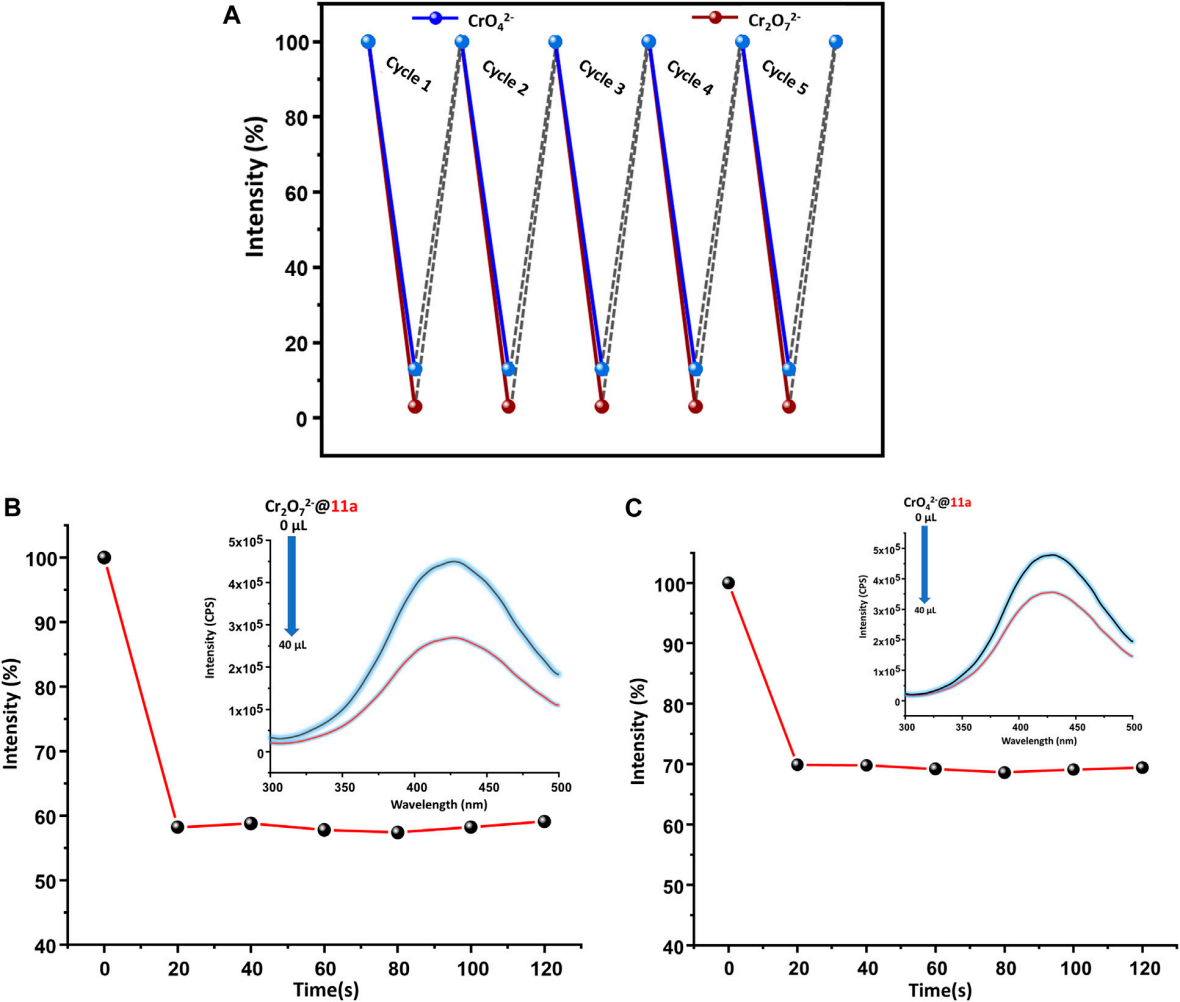
**(A)** Regeneration of pristine fluorescence intensity of **11a** towards CrO_4_^2−^ /Cr_2_O_7_^2−^ used for 5 cycles, Time dependent change in fluorescence intensity of **11a**, upon addition of **(B)** Cr_2_O_7_^2−^ /**(C)** CrO_4_^2−^ions (recorded up to 120 s after a regular interval of 20 s).

**FIGURE 7:**
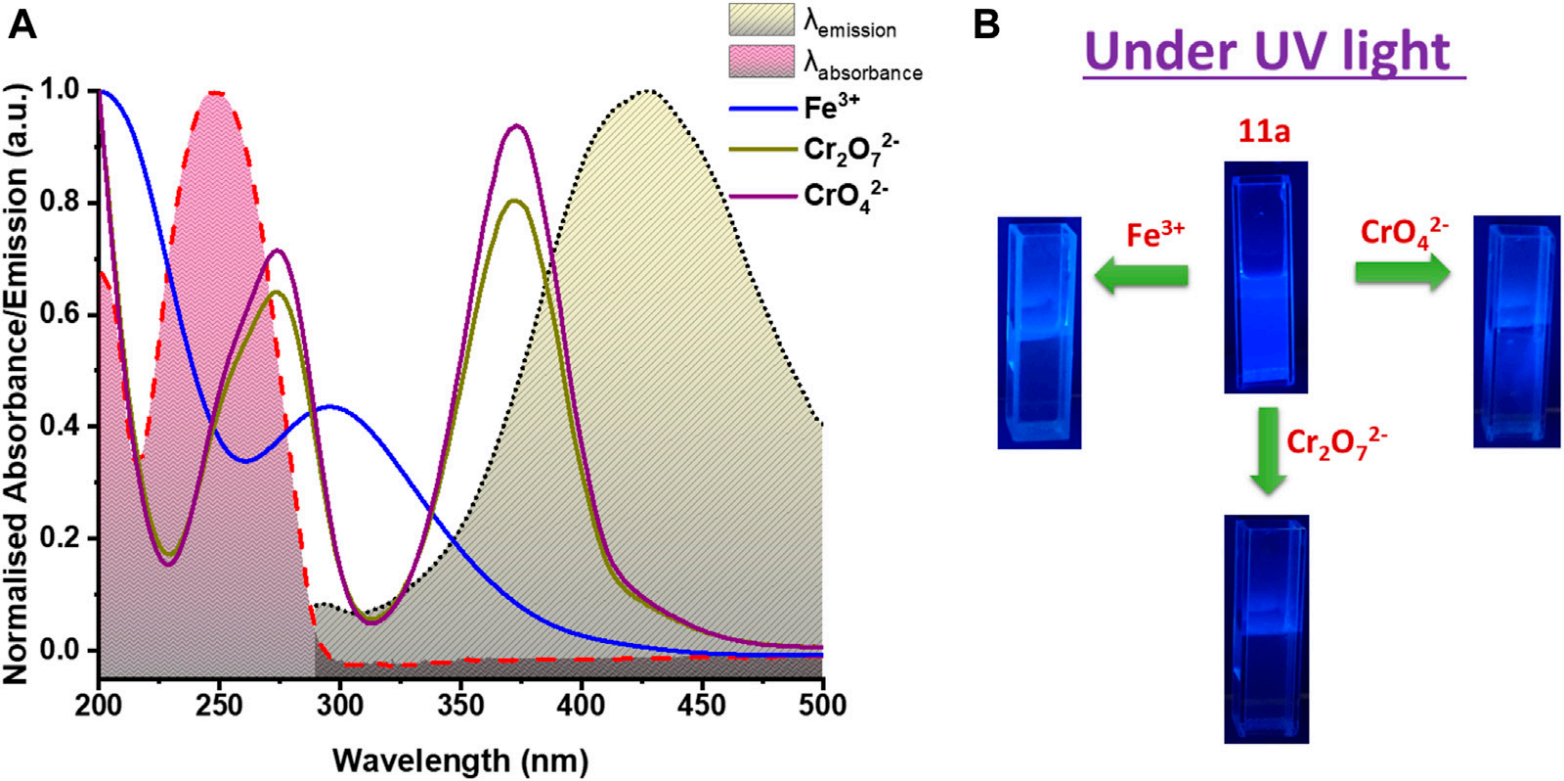
**(A)** Spectral overlap between absorbance spectra of studied analytes (Fe^3+^, Cr_2_O_7_^2 −^ and CrO_4_^2 −^) and emission as well as excitation spectrum of **11a**, **(B)** Visible change under UV light in luminescence intensity of **11a** on addition of Fe^3+^, Cr_2_O_7_^2−^ and CrO_4_^2−^ solution.

Given reclamation and reusability of a heterogeneous sensory material is of crucial importance, **11a** was centrifuged after sensing experiments, washed thoroughly with water, followed by methanol and finally air-dried. It was observed that PL intensity could be restored up to five sensing-recovery cycles (Figure 6A) and indicative of consistency of its performance towards hexavalent chromium ion detection. Structural robustness of **11a** during repetitive sensing was additionally assured from intact positions of all peaks in its PXRD pattern (Supplementary Figure S13) after sensing experiment. Further, fast-responsive analyte test (FRAT) was conducted to confirm the response time. Decrease in the intensity was recorded upon addition of 40 µl of Cr(VI) oxo anion solution to the MOF dispersion as a function of time. Emission response decreased to 69.9 and 58.2% for CrO_4_^2−^ and Cr_2_O_7_^2−^ ions (Figures 6B,C), respectively. Change in PL intensity was recorded up to 120 s after a regular interval of 20 s, which revealed no significant change in luminescence intensity beyond 20 s. These results further validated fast responsive sensing of both the Cr(VI) anionic species in water. Motivated by excellent solution-phase of detection, an attempt was made to visually detect Cr(VI) ions in the aqueous phase. MOF dispersion was exposed to UV light (265 nm), which resulted bright blue emission. Addition of just one drop of either CrO_4_^2−^ or Cr_2_O_7_^2−^ ions instantaneously diminished (Figure 7B) this emission and supports real-time visual monitoring as well.

### Luminescent Quenching Mechanism

To look into the sensing mechanism of Fe^3+^ ions and Cr(VI) oxo-anions, a series of experimental studies were performed. In all the cases, quenching of the PL intensity due to collapse of the framework was discarded on the basis of unaltered PXRD patterns (Supplementary Figure S13) after sensing. The possibility of exchange of Fe^3+^ ions with the constituting Cd(II) ions is nullified from fast-responsive detection, in a sense that trans metalation reaction in such a short time span is not possible (Figure 4A). Another probability includes adsorption of Fe^3+^ and/or Cr_2_O_7_^2−^/CrO_4_^2−^ ions within the framework. To this end, **11a** was separately immersed to aqueous solutions of Fe^3+^, Cr_2_O_7_^2−^and CrO_4_^2−^ for about a day. Resultant products Fe^3+^@**11a,** CrO_4_^2−^@**11a** and Cr_2_O_7_^2−^@**11a** were analysed by inductively coupled plasma optical emission spectrometry (ICP-OES), which validated no encapsulation of these ions even after prolonged exposure from the ratio of Cd (II) and other ions (Supplementary Table S2). Moreover, ICP-OES of the supernatant solution showed absence of Cd^2+^ ions and further corroborated no leaching of Cd(II) ions. Similarly, FT-IR analysis (Supplementary Figure S14) of the samples did not show any additional peak and eliminates the chances of encapsulation of these ions. As a matter of fact, the easy recyclability of **11a** after every detection through simple washing also indicates weak surface interaction between the studied analytes and the MOF.

In this milieu, the competition for the excitation energy absorption between **11a** and other ions may lead to the change of the luminescent signals. (Chen et al., 2017b; Dong et al., 2017). Figure 7A shows the UV absorption spectra of Fe^3+^, CrO_4_^2−^ and Cr_2_O_7_^2−^ plotted together with absorption and emission spectra of **11a**. For Fe^3+^, relatively broad and moderate adsorption was perceived covering both excitation and emission peak of **11a.** Of note, other metal ions have no such conspicuous absorption overlapping with the framework. This leads us to conclude that Fe^3+^ ions can absorb the excitation and emission light of **11a**, thereby decreasing its luminescence intensity. (Guo et al., 2019) On the other hand, competitive energy absorption was envisioned as a valid, alternative reason for optical response quenching. CrO_4_^2−^/Cr_2_O_7_^2−^ have two absorption bands in the range 220– 450 nm that cover a wide range of excitation and emission bands of **11a**. In contrast, other anions do not absorb in this particular wavelength region. So excitation energy will be strongly absorbed only by these hexavalent Cr(VI) anions, reducing UV-vis absorption of the **11a**, and resulting in substantial quenching. (Yi et al., 2015) Conversely, absorption spectra overlap of other metal ion and anions with absorption spectra of **11a** show no significant overlap (Supplementary Figure S15a, Supplementary Figure S15b) and validates selective detection of studied analytes. Indeed, the excellent extent of overlap between the absorption spectra of Fe^3+^, Cr(VI) oxo-ions and emission spectrum of **11a** helps in producing fluorescence quenching owing to the resonance energy transfer (RET).

## Conclusion

In conclusion, mixed-ligand approach has been effectively harnessed in synthesizing a hydrolytically robust, bipillar-layer Cd(II)-framework from the combination of bifunctional ligand 4-(4-carboxyphenyl)-1,2,4-triazole (H**L**) and linker *bpy*. The 2-fold interpenetrated structure with one-dimensional porous channels shows high open-air stability and retains its network integrity in common organic solvents. The π-electrons rich organic struts, suitably arranged through Cd(II) metal centres, allows high fluorescence intensity to the activated structure, which has been successfully utilized in sensitive and selective luminescent monitoring of Fe^3+^ ions in water with a 166 ppb limit of detection (LOD) and 20 s response time. The framework further reveals repetitive detection of two lethal Cr(VI) oxo-anions with fast-responsive emission quenching, where every quenching constants (CrO_4_^2−^: 1.73 × 10^4^ M^−1^; Cr_2_O_7_^2−^: 5.42 × 10^4^ M^−1^) and LOD values (CrO_4_^2−^: 179 ppb; Cr_2_O_7_^2−^: 114 ppb) rank among the best sensory MOFs for detection of Cr(VI) ions in water. Apart from solution-titration based turn-off responses, visual detection of these ions under UV light has been observed that validates real-time applicability of the material. Owing to selective, multi-cyclic, fast responsive and aqueous phase luminescent based detection of contaminating ions, this material promises its suitability for sustainable applications. Detailed experimental studies have been carried out to understand the mechanism of selective quenching, including competitive absorption of the excitation energy and the resonance energy transfer between the host framework and individual analytes. Given aqueous phase acute luminescent detection of water contaminating ions belongs to important global agendas for sustainability, this robust MOF highlights the importance of structure–property synergies and represents a futuristic material for sensing applications.

## Experimental Section

### Synthesis of 4- (1H-imidazol-1-yl) benzoic acid (HL)

The ligand was synthesised and characterised by previously reported method (**Refer to SI**).

### Synthesis of CSMCRI-11

A mixture of Cd(NO_3_)_2_∙4H_2_O (48.4 mg, 0.156 mmol), 4,4ʹ-bipyridyl (31.25 mg, 0.2 mmol) and 4-(1H-imidazol-1-yl)benzoic acid (HL) (37.23 mg, 0.19 mmol) was dissolved in N,N-dimethylformamide (DMF; 7 ml) sealed in 15 ml glass vial, and heated at 120 °C for 2 days. The colourless, rectangular crystals were isolated (Supplementary Scheme S2) in 65% yield.

Anal.Calcd. for C_33_H_33_N_8_O_10_Cd_1.5_ = [Cd_1.5_ **(*L*)**_2_ *(bpy)(*NO_3_)]·DMF·2H_2_O: C, 45.54; H, 3.82; N, 12.88%; found: C, 44.23; H, 3.92; N, 12.43%. FT-IR (KBr) analysis: 1303, 1399, 1563, 1612, 1663, & 3414 cm^−1^ (Supplementary Figure S4).

### Synthesis of 11a

As synthesised crystals were washed with fresh DMF and finally dried in air. The guest solvents in **CSMCRI-11** were exchanged with methanol by soaking the crystals in methanol for 3 days followed by exchanging the solvent 3 times a day. The crystals were dried overnight under vacuum at 120 °C to generate a solvent-free framework **11a**.

Anal.Calcd. for C_30_H_22_N_7_O_7_Cd_1.5_ = [Cd_1.5_(***L***)_2_(*bpy*)(NO_3_)] C, 47.34; H, 2.91; N, 12.88%. found: C, 46.17; H, 3.01; N, 12.45%.

## Data Availability

The datasets presented in this study can be found in online repositories. CCDC 2054352 contains the supplementary crystallographic data for this paper. The data can be obtained free of charge from The Cambridge Crystallographic Data Centre via www.ccdc.cam.ac.uk/data_request/cif.
