# Supplementary material for: Devising Mixed-Ligand Based Robust Cd(II)-Framework From Bi-Functional Ligand for Fast Responsive Luminescent Detection of Fe3+ and Cr(VI) Oxo-Anions in Water With High Selectivity and Recyclability
Source: Front Chem. 2021 May 5;9:651866. doi: 10.3389/fchem.2021.651866 (PMC8131680; doi:10.3389/fchem.2021.651866)
Supplement: Supplementary file 1 [file Table1.DOCX]

**Table 1*.* Performance Characteristics of 11a for Detection of Fe^3+^, CrO_4_^2−^ and Cr_2_O_7_^2−^ Ions in Water**

| **Analyte** | **Fe^3+^** | **Cr_2_O_7_^2−^** | **CrO_4_^2−^** |
| --- | --- | --- | --- |
| Sensing Mode | Turn-off | Turn-off | Turn-off |
| Quenching extent (%) | 91.60 | 96.13 | 87.15 |
| K_SV_ (M^−1^) | 1.91 × 10^4^ | 2.18 × 10^4^ | 1.46 × 10^4^ |
| LOD (ppb) | 166 ppb | 114 ppb | 179 ppb |
| Ultra-fast nature | 40 µL / 20 sec | 40 µL /20 sec | 40 µL / 20 sec |
