# Supplementary material for: Devising Mixed-Ligand Based Robust Cd(II)-Framework From Bi-Functional Ligand for Fast Responsive Luminescent Detection of Fe3+ and Cr(VI) Oxo-Anions in Water With High Selectivity and Recyclability
Source: Front Chem. 2021 May 5;9:651866. doi: 10.3389/fchem.2021.651866 (PMC8131680; doi:10.3389/fchem.2021.651866)
Supplement: Supplementary file 2 [file DataSheet1.pdf]

Supporting Information

Devising Mixed-Ligand Based Robust Cd(II)-Framework From Bi-Functional Ligand for Fast Responsive Luminescent Detection of Fe^{3+} and Cr(VI) Oxo-Anions in Water With High Selectivity and Recyclability

Manpreet Singh,[1,2] Gaurav Kumar,[1,2] and Subhadip Neogi*[1,2]

[1]Inorganic Materials & Catalysis Division, CSIR-Central Salt and Marine Chemicals Research Institute (CSMCRI), Bhavnagar, Gujarat 364002, India
[2]Academy of Scientific and Innovative Research (AcSIR), Ghaziabad- 201002, India
*E-mail: sneogi@csmcri.res.in

S. No.	Content	Page No.
1.	Physical measurements	S3
2.	Chemicals	S3
3.	Single Crystal X-ray Crystallography	S3
4.	**Figure S1.** (a) Asymmetric unit of **CSMCRI-11** and (b) demonstration of π–π stacking interactions between the benzene rings of ligand in the structure.	S4
5.	Synthesis of ligand and **CSMCRI-11**	S4-S6
6.	**Figure S2.** (a) ESI-MS spectrum of 4-(1H-imidazol-1-yl) benzoic acid **(HL)**, (b)^{1}H-NMR spectrum of -(1H-imidazol-1-yl) benzoic acid **(HL)**.	S5
7.	Synthetic scheme of the MOF $[Cd_{1.5}(L)_2(bpy)(NO_3)]\cdot DMF\cdot 2H_2O$ **(CSMCRI-11)**	S6
8.	**Figure S3.** SEM image of **CSMCRI-11**	S6
9.	**Figure S4.** FT-IR spectra (KBr pellets, cm^{-1}) of **CSMCRI-11** (black) and **11a** (red).	S6
10.	**Figure S5.** Thermogravimetric analysis of as-made **CSMCRI-11** (a) and **11a** (b)	S7
11.	**Figure S6.** PXRD pattern of **11a** after exposure to water for 10 days.	S7
12.	**Figure S7.** (a) UV-Vis spectra of constituting ligands of **CSMCRI-11**, and **11a** (b) Emission intensity of ligand HL, *bpy* linker, and **11a** (all dispersed in H_2O). (c) Emission intensity of **11a** dispersed in various solvents**.** (d) Emission spectra of **11a** in water dispersion and supernatant solution after removal of **11a** (red line). (e) UV-Vis spectra of **11a** in different solvents.	S8

**13.**	**Figure S8.** Fluorescence quenching efficiency of **11a** (1 mg/ 50 mL water) for different Cations. Quenching efficiency is calculated using equation $(I_0 - I)/I_0 \times 100\%$, where I and I_0 denotes the emission intensities after and before the addition of studied analytes, respectively.	S9
**14.**	**Figure S9.** Linear region of fluorescence intensity of **11a** upon addition of Fe^{3+} (0 – 120 μL, 10 μM stock solution).	S9
**15.**	**Figure S10.** Fluorescence quenching efficiency of **11a** (1 mg/ 2 mL water) for different Anions. Quenching efficiency of **11a** is calculated using equation $(I_0 - I)/I_0 \times 100\%$, where I and I_0 denotes the emission intensities after and before the addition of studied analytes, respectively.	S10
**16.**	**Figure S11.** Linear region of fluorescence intensity of **11a** upon addition of $Cr_2O_7^{2-}$ (0 – 120 μL, 10 μM stock solution).	S10
**17.**	**Figure S12.** Linear region of fluorescence intensity of **11a** upon addition of CrO_4^{2-} (0 – 120 μL, 10 μM stock solution).	S11
**18.**	**Figure S13.** PXRD curves of **11a** after five sensing recovery cycles for Fe^{3+}, $Cr_2O_7^{2-}$, CrO_4^{2-} (2.5 mM), showing that structural integrity of the framework is maintained.	S11
**19.**	**Figure S14.** FTIR spectra of **11a**, Fe^{3+}@**11a**, $Cr_2O_7^{2-}$@**11a**, CrO_4^{2-}@**11a.**	S12
**20.**	**Figure S15.** Spectral overlap between absorbance spectra of (a) cations (b) anions and absorption spectra/emission spectra of **11a** in water	S12
**21.**	**Figure S16.** Change UV pattern upon adsorption of (a) Fe^{3+} (b) $Cr_2O_7^{2-}$ (c) CrO_4^{2-} up to 120 minutes.	S13
**22.**	**Table S1.** Crystal structure and refinement parameters	S13
**23.**	ICP analysis results and analysis	S14
**24.**	**Table S2.** ICP analysis results for samples	S14
**25.**	**Table S3.** Calculation of standard deviation of fluorescence intensity and limit of Detection for **11a** towards Fe^{3+}	S14
**26.**	**Table S4.** Calculation of standard deviation of fluorescence intensity and limit of detection for **11a** towards $Cr_2O_7^{2-}$	S15
**27.**	**Table S5.** Calculation of standard deviation of fluorescence intensity and limit of detection for **11a** towards CrO_4^{2-}	S15
**28.**	**Table S6.** A comparison of quenching constants and corresponding LODs for various luminescent MOFs used for detection of Fe^{3+}	S16-S17
**29.**	**Table S7.** A comparison of quenching constants and corresponding LODs for various luminescent MOFs used for detection of $Cr_2O_7^{2-}$/CrO_4^{2-}	S17-S18

Physical measurements

The infrared spectra (IR) of the samples were recorded using the KBr pellet method on a Perkin–Elmer GX FTIR spectrometer in the region of 400-4000 cm^{-1}. Powder X-ray diffraction (PXRD) data were collected using a PANalytical Empyrean (PIXcel 3D detector) System equipped with Cu Kα (λ=1.54 Å) radiation. Microanalyses of the compounds were Conducted using elementary vario MICRO CUBE analyser. Thermogravimetric analyses (TGA) (heating rate of 5 °C/min under N_2 atmosphere) were performed with a Mettler Toledo Star SW 8.10 system. The solvent-exchanged (methanol-exchanged) frameworks were then degassed overnight under vacuum at 120 °C to generate **11a**. UV-Vis spectra recorded using Shimadzu UV-3101 PC spectrometer and the luminescence experiments were performed at room temperature using a Fluorolog Horiba Jobin Yvon spectrophotometer.

Chemicals

Analytical grade cadmium nitrate hexahydrate $Cd(NO)_3·4H_2O$ (AR) and 4,4′-bipyridine were purchased from Tokyo Chemical Industries private limited. All the solvents such as N, N′-dimethylformamide (DMF) (Fisher Scientific), methanol (S. D. Fine Chemicals, India), were purchased and used without any further purification. Ligand (1H-imidazol-1-yl)benzoic acid was synthesised as mentioned in SI. All the metals salts used for the sensing experiments were procured commercially and used with any further analysis.

Single Crystal X-ray Crystallography

Single crystals with suitable dimensions were chosen under an optical microscope and mounted on a glass fibre for data collection. Intensity data for as synthesized colorless crystals of **CSMCRI-11** were collected using graphite-monochromated $MoK_α$ (λ=0.71073 Å) radiation on a Bruker SMART APEX diffractometer equipped with CCD area detector at 173 K, The linear absorption coefficients, scattering factors for the atoms, and the anomalous dispersion corrections were taken from International Tables for X-ray Crystallography. The data integration and reduction were performed with SAINT[1] software. Absorption corrections to the collected reflections were accounted with SADABS[2] using XPREP.[3] The structure was solved by direct method using SIR-97[4] and was refined on F^2 by the full-matrix least-squares technique using the SHELXL-2014[5] program package. All H atoms were placed in calculated positions using idealized geometries (riding model) and assigned fixed isotropic displacement parameters using the SHELXL default. To give an account of disordered electron densities associated with solvent molecules, the "SQUEEZE" protocol in PLATON[6] was applied that produced a set of solvent free diffraction intensities. Final cycles of least-squares refinements improved both the R values and Goodness of Fit with the modified data set after subtracting the contribution from the disordered solvent molecules, using SQUEEZE program. The crystal and refinement data for solvent free **CSMCRI-11** is listed in Table S1.

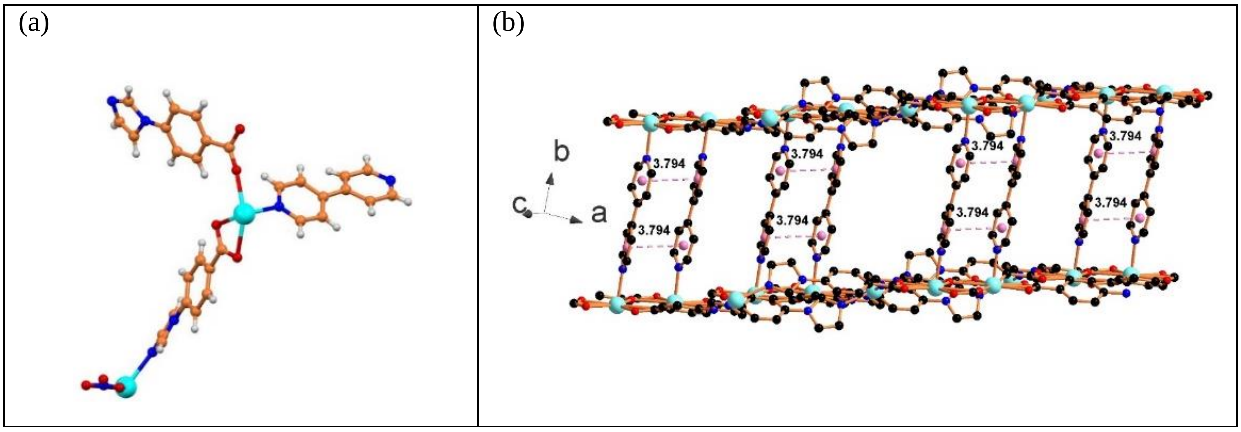

Figure S1. (a) Asymmetric unit of **CSMCRI-11** and (b) demonstration of π–π stacking interactions between the benzene rings of ligand in the structure.

Synthesis of ligand and CSMCRI-11

The ligand **4-(1H-imidazol-1-yl) benzoic acid** was prepared by slight modification of the following known procedure.[1]

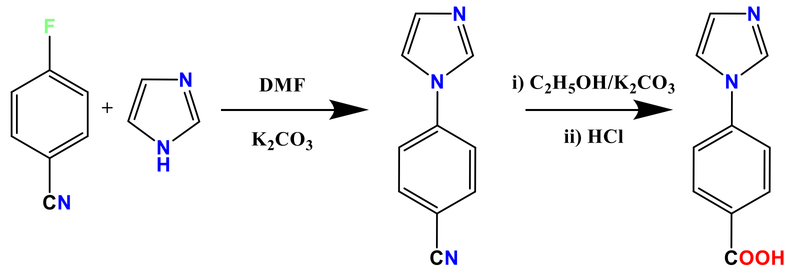

Scheme S1. Synthetic scheme of ligand.

In a round bottom flask 8g of K_2CO_3(activated), 2.1g of imidazole and 3.63g of 4-fluorobenzonitrile were dissolved in anhydrous DMF. Solution was stirred under N_2 atmosphere and heated at 130 °C for 24 h. Afterwards, mixture was poured in ice cold water and allowed to stand for 24 h. The white ppt were filtered and was dissolved in ethanol (50ml) followed by dropwise addition of 6 N KOH solution (50 ml) and it was refluxed at 80 °C for 12 hours.

Reaction was cooled and resulting solution was acidified (pH 4.0) by 3 N HCl under ice-cold condition, which yields white precipitate. This was separated by filtration and washed several times with water. Finally, the white crude solid was dried at room temperature, followed by overnight vacuum drying. Ligand was characterised by LCMS (fig. 1a) and NMR (fig. 1b).

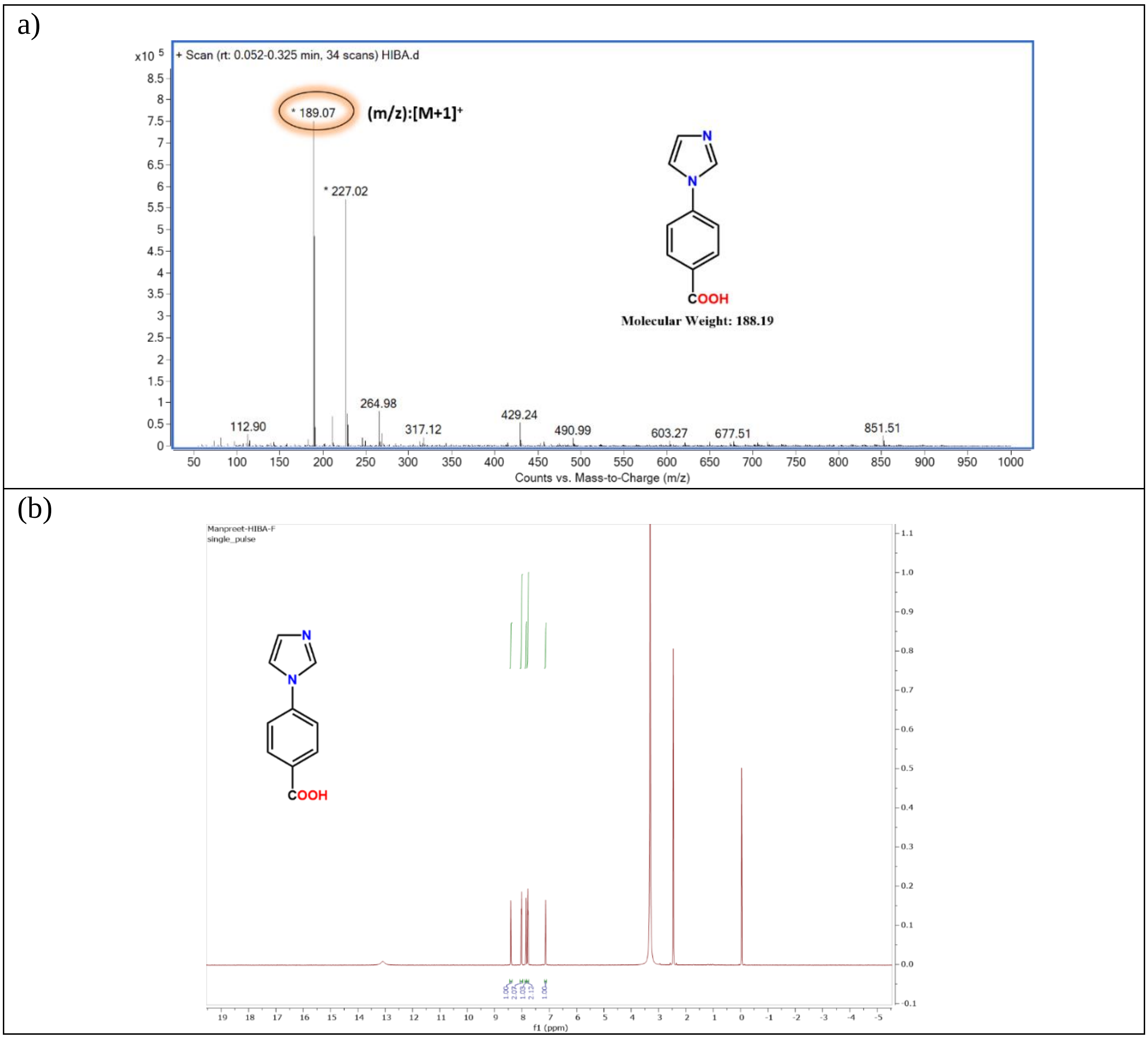

Figure S2. **(a)** ESI-MS spectrum of 4-(1H-imidazol-1-yl) benzoic acid **(HL)**, (b)^{1}H-NMR spectrum of -(1H-imidazol-1-yl) benzoic acid (**HL**).

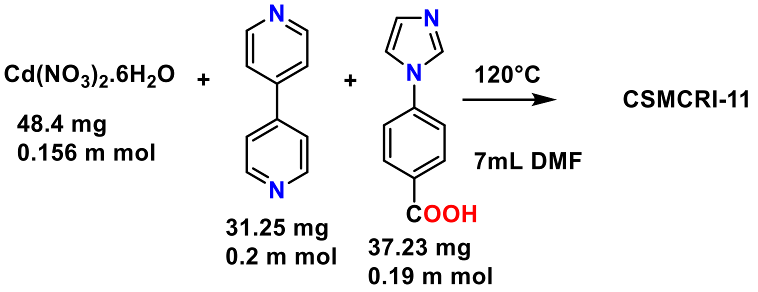

Scheme S2. Synthetic scheme of the MOF $[Cd_{1.5}(\mathbf{L})_2(bpy)(NO_3)]\cdot DMF\cdot 2H_2O$ (**CSMCRI-11**).

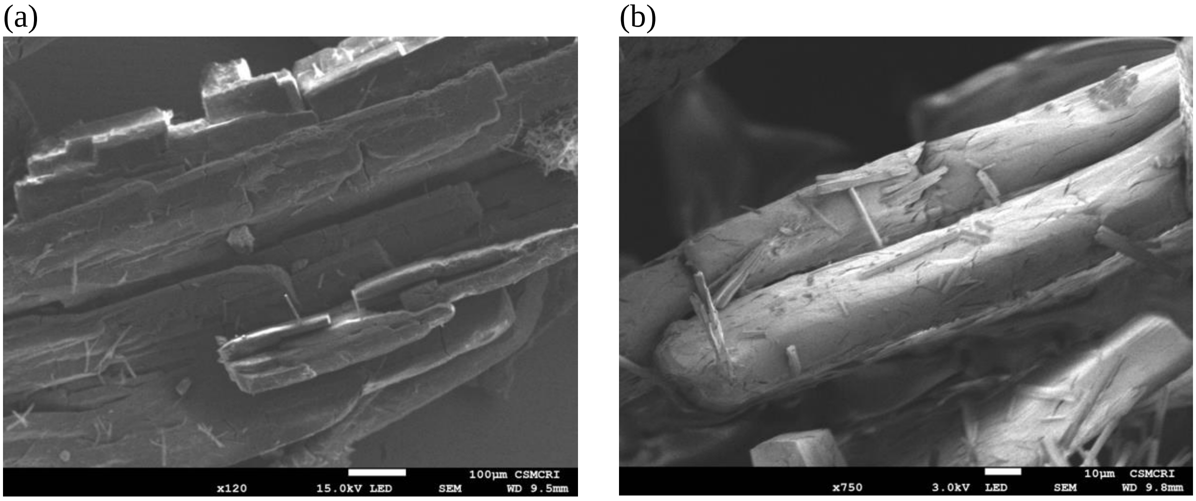

Figure S3. SEM images of as-synthesised **CSMCRI-11**.

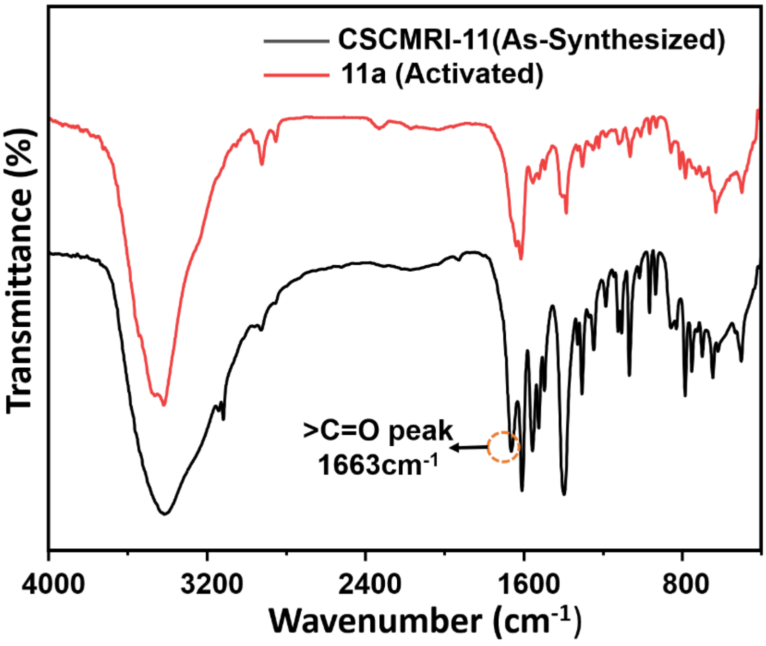

Figure S4. FT-IR spectra (KBr pellets, cm^{-1}) of **CSMCRI-11** (black) and **11a** (red).

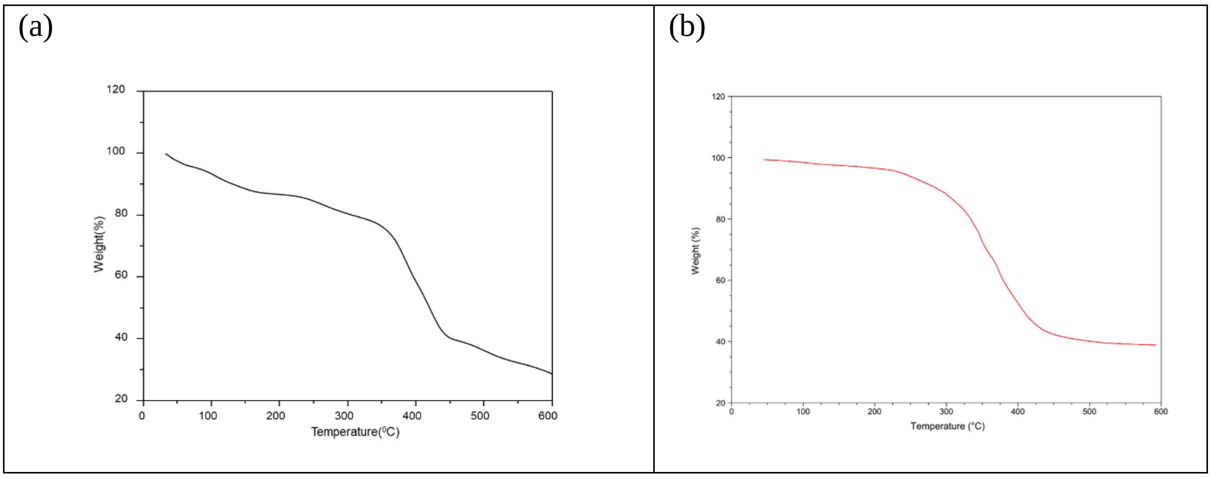

Figure S5. Thermogravimetric analysis of as-made **CSMCRI-11** (a) and **11a** (b).

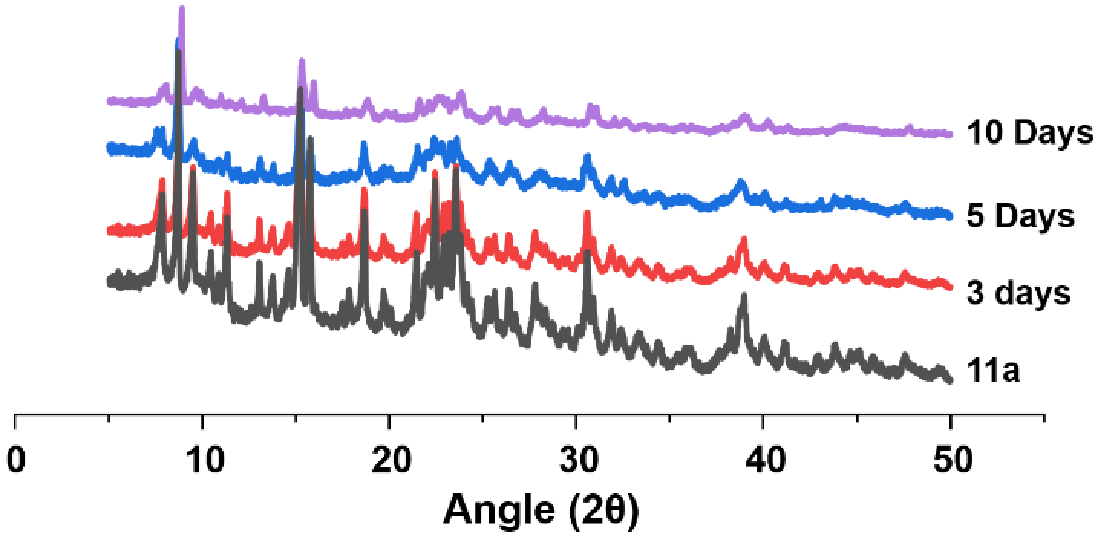

Figure S6. PXRD pattern of **11a** after exposure to water for 10 days.

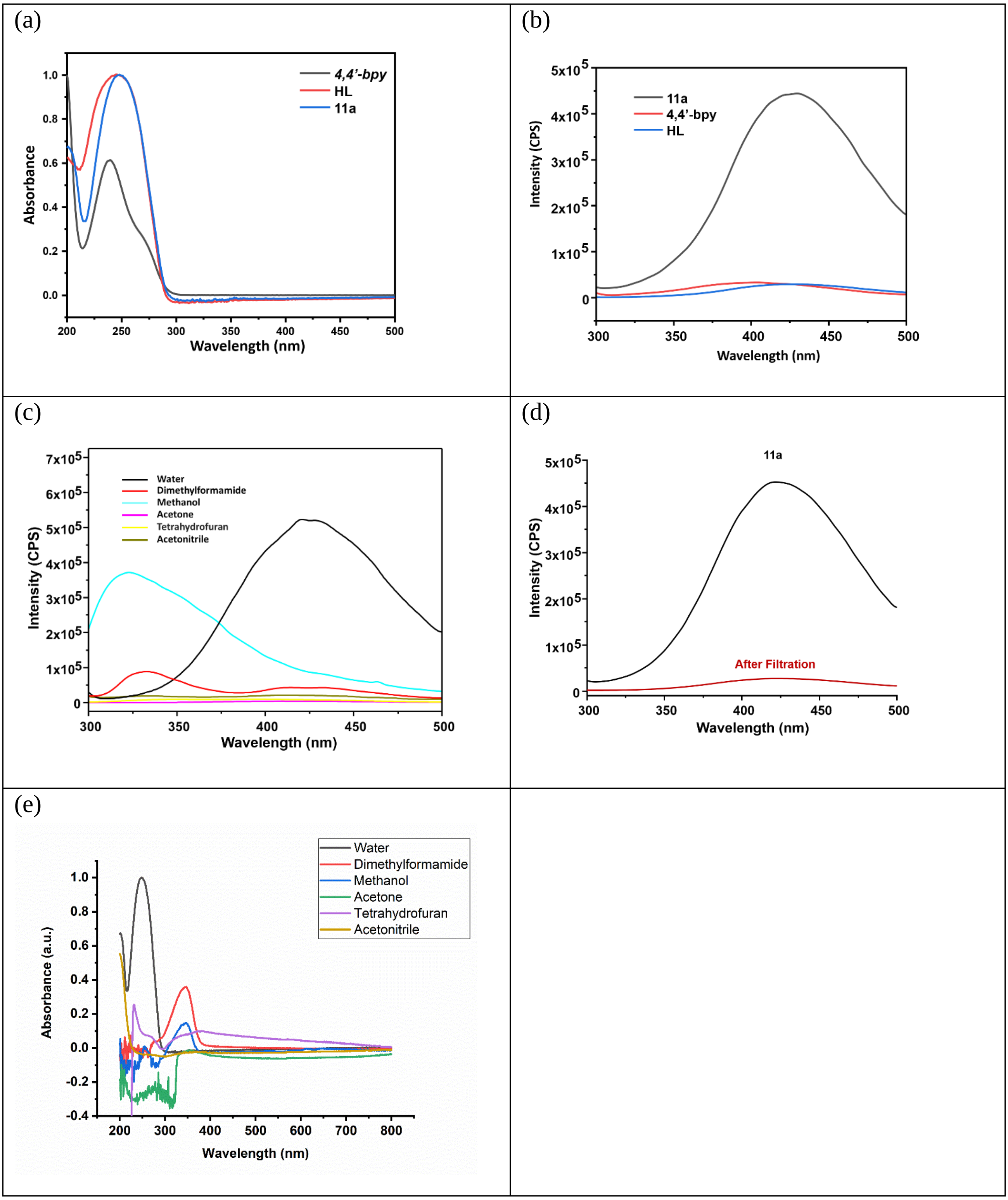

Figure S7. (a) UV-Vis spectra of constituting ligands of **CSMCRI-11**, and **11a** (b) Emission intensity of ligand H**L**, *bpy* linker, and **11a** (all dispersed in H_2O). (c) Emission intensity of **11a** dispersed in various solvents**.** (d) Emission spectra of **11a** in water dispersion and supernatant solution after removal of **11a** (red line). (e) UV-Vis spectra of **11a** in different solvents

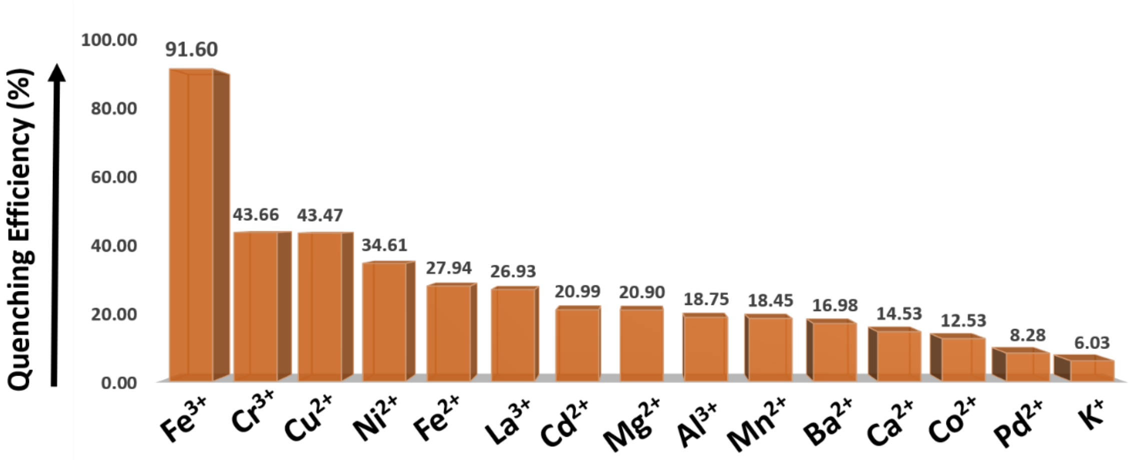

Figure S8. Fluorescence quenching efficiency of **11a** (1 mg/ 50 mL water) for different Cations. Quenching efficiency is calculated using equation $(I_0 - I)/I_0 \times 100\%$, where I and I_0 denotes the emission intensities after and before the addition of studied analytes, respectively.

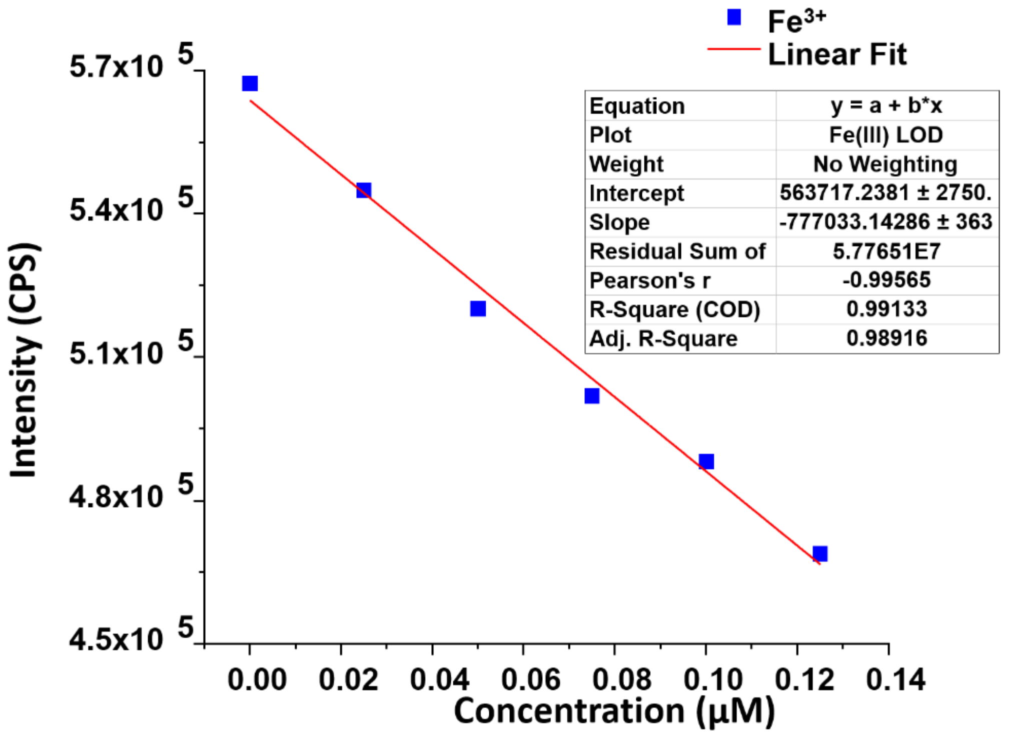

Figure S9. Linear region of fluorescence intensity of **11a** upon addition of Fe^{3+} (0 – 120 μM)

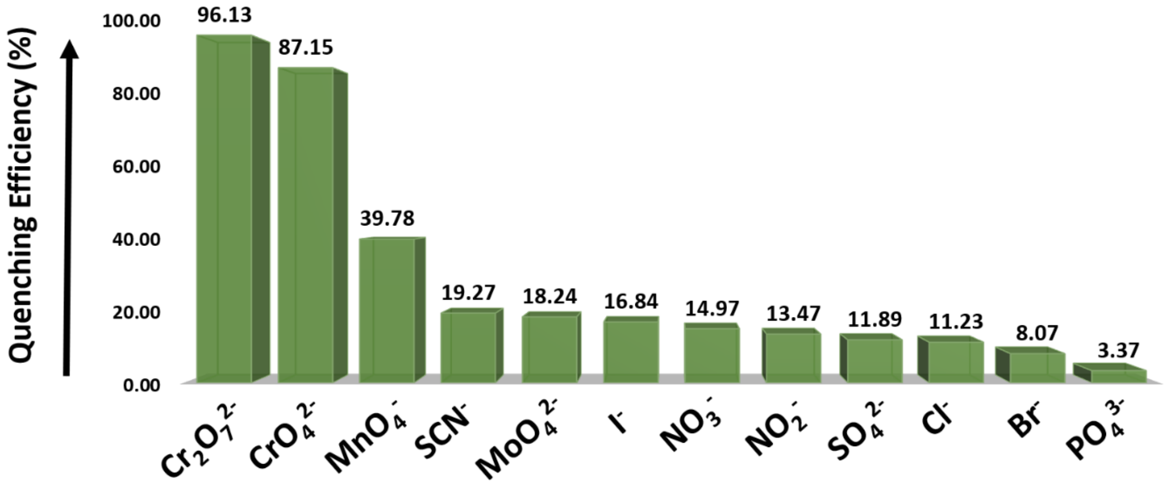

Figure S10. Fluorescence quenching efficiency of **11a** (1 mg/ 50 mL water) for different Anions. Quenching efficiency of **11a** is calculated using equation $(I_0 - I)/I_0 \times 100\%$, where I and I_0 denotes the emission intensities after and before the addition of studied analytes, respectively.

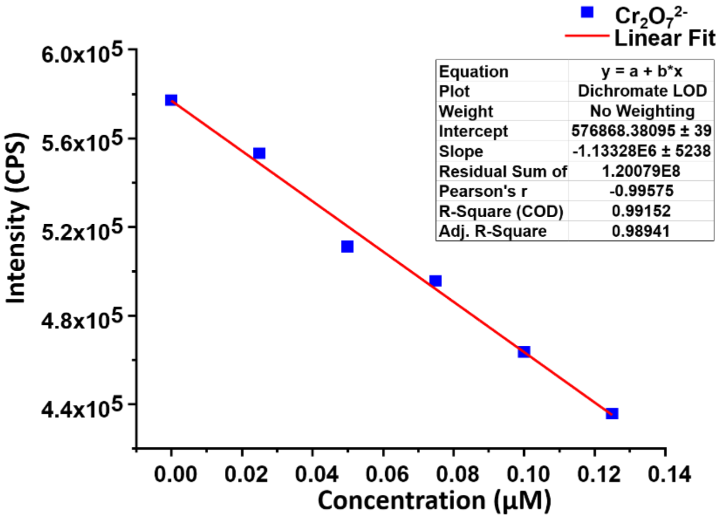

Figure S11. Linear region of fluorescence intensity of **11a** upon addition of $Cr_2O_7^{2-}$ (0 – 120 µL, 10 µM stock solution).

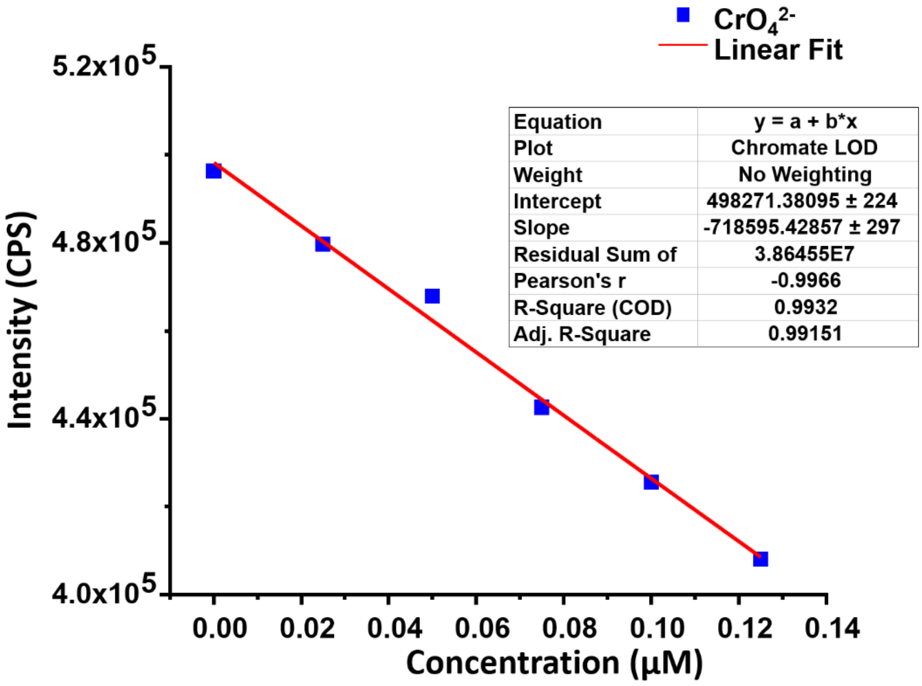

Figure S12. Linear region of fluorescence intensity of **11a** upon addition of CrO_4^{2-} (0 – 120 μL, 10 μM stock solution).

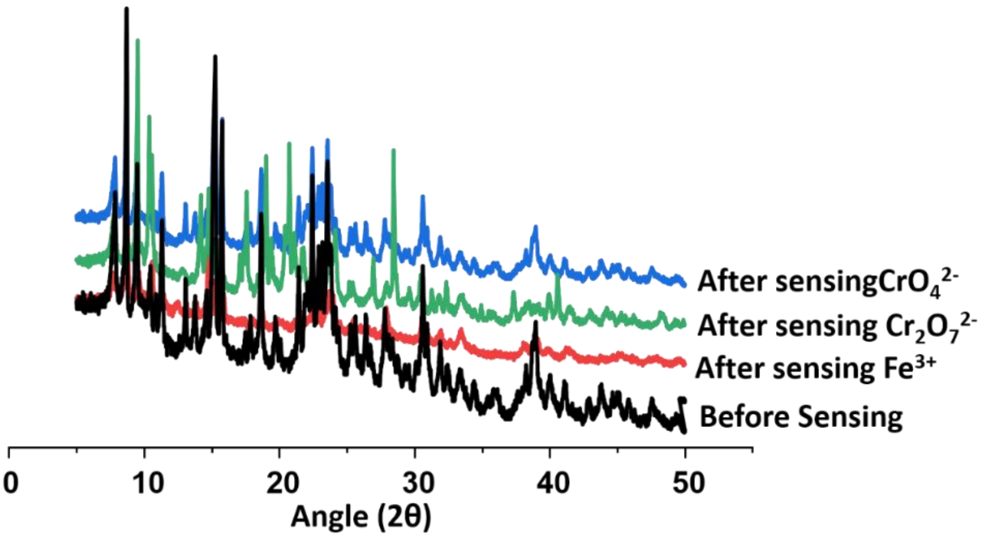

Figure S13. PXRD curves of **11a** after five sensing recovery cycles for Fe^{3+}, $Cr_2O_7^{2-}$, CrO_4^{2-} (2.5 mM), showing that structural integrity of the framework is maintained.

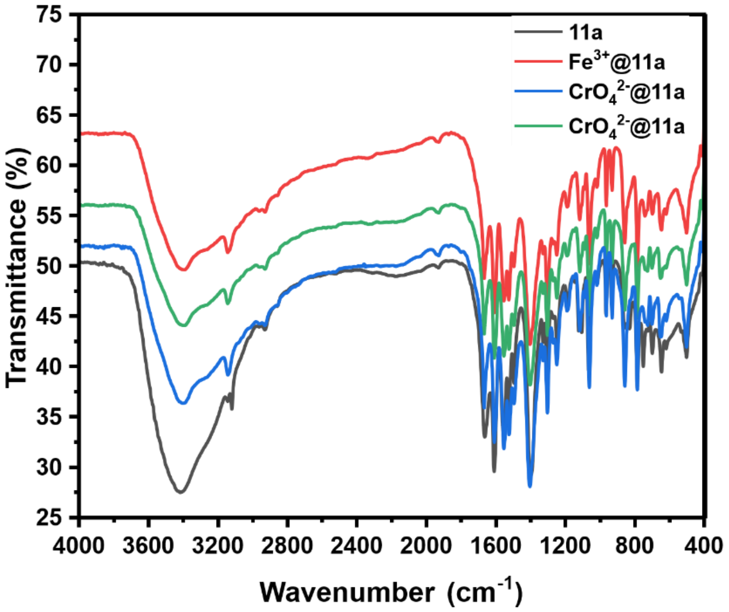

Figure S14. FTIR spectra of **11a**, Fe^{3+}@**11a**, CrO_4^{2-}@**11a**, $Cr_2O_7^{2-}$@**11a.**

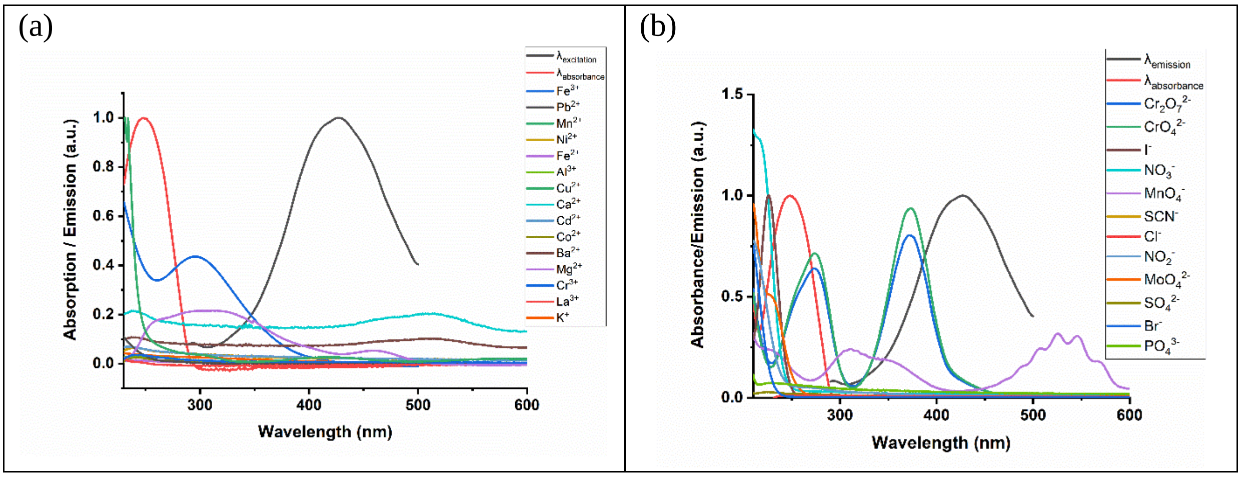

Figure S15. Spectral overlap between absorbance spectra of (a) cations (b) anions and absorption spectra/emission spectra of 11a in water.

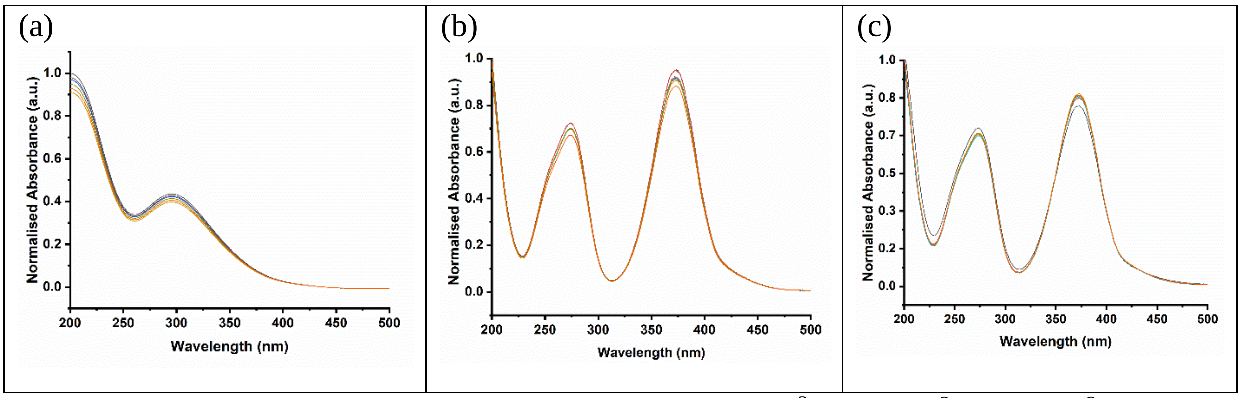

Figure S16. Change in UV pattern upon adsorption of (a) Fe^{3+} (b) CrO_4^{2-} (c) $Cr_2O_7^{2-}$ up to 120 minutes.

Table S1. Crystal data and structure refinement for **CSMCRI-11**

Identification code	**CSMCRI-11**
Empirical formula	$C_{30}H_{22}Cd_{1.5}N_7O_7$
Formula weight	761.17
Temperature/K	273.15
Crystal system	monoclinic
Space group	C2/c
a/Å	41.129(8)
b/Å	11.649(2)
c/Å	16.487(3)
α/°	90
β/°	100.533(7)
γ/°	90
Volume/Å^3	7766(2)
Z	4
ρ_{calc}g/cm^3	0.651
μ/mm^{-1}	0.437
F(000)	3023.7
Crystal size/mm^3	0.18 × 0.16 × 0.14
Radiation	Mo Kα (λ = 0.71073)
2Θ range for data collection/°	4.02 to 56.88
Index ranges	$-55 \le h \le 54$, $-15 \le k \le 15$, $-22 \le l \le 22$
Reflections collected	52513
Independent reflections	9692 [R_{int} = 0.0853, R_{sigma} = 0.0724]
Data/restraints/parameters	9692/0/408
Goodness-of-fit on F^2	1.008
Final R indexes [I>=2σ (I)]	R_1 = 0.0706, wR_2 = 0.1287
Final R indexes [all data]	R_1 = 0.0912, wR_2 = 0.1377
Largest diff. peak/hole / e Å^{-3}	1.39/-1.33

ICP analysis results and analysis:

10 mg of **11a** was centrifuge after sensing experiment and supernated was collected and heated till dryness. Solid was again dissolved in freshly prepared aqua regia (3:1 (v/v) conc. HCl: HNO_3) (4 mL) and heated to 140 °C till dryness. Same process was repeated thrice to ensure complete digestion. Residues were solubilized in 10 ml H_2O and the obtained solutions were used for ICP analysis after required dilution. No trace of leached cadmium was observed and ICP results before and after sensing were found to be same confirming no encapsulation of the ions inside the framework.

Table S2. ICP analysis results for samples

Entry	Sample Code	After Analysis		Ratio (Cd:Others)
		Cd	Others	
**1.**	**Fe^{3+}@11a**	**2.08**	**0.31**	**6.7:1**
**2.**	**$Cr_2O_7^{2-}$@11a**	1.92	0.33	5.8:1
**3.**	**CrO_4^{2-}@11a**	2.14	0.35	6.11:1
**4.**	**Superannuate Solution**	0.0	--	00

Table S3. Calculation of standard deviation of fluorescence intensity and limit of Detection for **11a** towards Fe^{3+}

Blank Reading (**11a**)	Fluorescence Intensity (CPS)
Reading 1	540677
Reading 2	460677
Reading 3	470677
Reading 4	466594
Reading 5	588271
Standard Deviation(σ)	56621.12305
The slop of the graph(K)	777033 μM^{-1}
Detection Limit(3σ/K)	0.219 µM
Limit of detection (Fe^{3+})	0.166 ppm
Limit of detection (Fe^{3+})	166 ppb

Table S4. Calculation of standard deviation of fluorescence intensity and limit of detection for **11a** towards $Cr_2O_7^{2-}$

Blank Reading (**11a**)	Fluorescence Intensity (CPS)
Reading 1	540677
Reading 2	460677
Reading 3	470677
Reading 4	466594
Reading 5	588271
Standard Deviation(σ)	56621.12305
The slop of the graph(K)	1133280 μM^{-1}
Detection Limit(3σ/K)	0.150 µM
Limit of detection ($Cr_2O_7^{2-}$)	0.114 ppm
Limit of detection ($Cr_2O_7^{2-}$)	114 ppb

Table S5. Calculation of standard deviation of fluorescence intensity and limit of detection for **11a** towards CrO_4^{2-}

Blank Reading (**11a**)	Fluorescence Intensity (CPS)
Reading 1	540677
Reading 2	460677
Reading 3	470677
Reading 4	466594
Reading 5	588271
Standard Deviation(σ)	56621.12305
The slop of the graph(K)	718695 μM^{-1}
Detection Limit(3σ/K)	0.236 µM
Limit of detection (CrO_4^{2-})	0.179 ppm
Limit of detection (CrO_4^{2-})	179 ppb

Table S6. A comparison of quenching constants and corresponding LODs for various luminescent MOFs used for detection of Fe^{3+}.

Entry	Fluorescence Material	Medium	K_{sv} (M^{-1})	Detection Limit	Reference
1	$\{Eu_2(L_1)_2(HCOO)_2(H_2O)_6\}_n$	DMF	1.58×10^3	3.3×10^{-7} M	*Dalton Trans.* **2013**, *42*, 12403-12409
2	$Eu(L_2)_3$	Water	4.1×10^3	5×10^{-4} M	*ACS Appl. Mater. Interfaces 2013, 5, 3, 1078-1083*
3	$[Ni(L_3)_2(L_4)_2(H_2O)_2]$	DMF		4.8×10^{-5} M	*Inorg. Chem.* **2017**, *56*, 2936-2940
	$[Cd_2(L_3)_2(L_4)(H_2O)]$	DMF		3.6×10^{-5} M	
4	$[Eu(L_5)_2(NO_3)]\cdot H_2O$	ethanol		2.6×10^{-5} M	*Inorg. Chem.* **2016**, *55*, 3952-3959
5	$\{[Eu(L_6)(H_2O)_2]\cdot NMP\cdot H_2O\}_n$	DMF	383395.8		*Inorg. Chem.* **2016**, *55*, 10114-10117
6	$[Eu(Hpzbc)_2(NO_3)]\cdot H_2O$	Ethanol		2.6×10^{-5} M	*Inorg. Chem.* **2016**, *55*, 3952-3959
7	$\{[Cd(5\text{-}asba)(bimb)]\}_n$	Water	1.78×10^4		*J. Mater. Chem. C* **2016**, *4*, 11404-11418
8	$[Tb_3(TCA)_2(DMA)_{0.5}(OH)_3(H_2O)_{0.5}]\cdot 3H_2O$	Water Methanol	3.714×10^4	8×10^{-6} M	*RSC Adv.* **2016**, *6*, 94622-94628
9	$\{[Cd(L_7)(BPDC)]\cdot 2H_2O\}_n$	Water	3.63×10^4	2.21×10^{-6} M	*Cryst. Growth Des.* **2017**, *17*, 67-72
10	$\{[Cd(L_8)(L_9)]\cdot 2H_2O\}_n$	DMF	5.57×10^4	2.5×10^{-6} M	*Dalton Trans.* **2017**, *46*, 2332-2338
11	534-MOF-Tb(L_{10})	Water	5.51×10^3	0.13 mM	*J. Mater. Chem. C.* **2017**, *5*, 2015-2021
12	$[Eu(HL_{11})(DMF)(H2O)2]\cdot 3\ H2O$	Water	1519		*Chem. Eur. J.* **2015**, *21*, 15806-15819
	$[Tb(HL_{11})(DMF)(H_2O)_2]\cdot 3H_2O$	Water	4749	5×10^{-5} M	
	$[Cd_4(L_{11})_2(DMF)_4(H_2O)_2].3H_2O$	Water			
	$[Zn_3(HL_{11})_2(H_2O)_2]\cdot 2DMF\cdot 7H_2O$	Water	381.85		
13	Al-MIL-53-N_3	water	6.13×10^3	3×10^{-8} M	*Dalton Trans.* ***2018****, 47, 2690–2700*
14	BUT-15	Water	1.66×10^4	3×10^{-7} M	*ACS Appl. Mater. Interfaces* ***2017****, 9, 10286-10295*
15	$\{[Eu(Pyridine\text{-}Carboxylate,)(BPDC)_{1/2}(NO_3)]\cdot H_2O\}_n$	DMF	5.16×10^4		*ACS Appl. Mater. Interfaces* **2017**, *9*, 1629-1634
	$\{[Tb(Pyridine\text{-}Carboxylate,)(BPDC)_{1/2}(NO_3)]\cdot H_2O\}_n$	DMF	4.30×10^4		
16	$Cd_2(bptc)(2,2'\text{-}bipy)_2(H_2O)_2$	Water	1.9×10^4	1.02×10^{-5} M	*Inorg. Chem.* ***2017****, 56, 11768-11778*
17	$La(TPT)(DMSO)_2$	ethanol	1.36×10^4		*Dalton Trans. 2015, 44, 13340-13346*
18	$[Zn_2(TPOM)(NDC)_2]3.5H_2O$	water	1.9×10^4	2×10-6 M	*Inorg. Chem.* ***2017****, 56, 12348-12356*
19	$[Zr_6O_4(OH)_4(C_{20}H_{10}O_4S_2)_6].2.5DMF.11H_2O$	Water	9.1×10^3	1.8×10^{-4} M	*Dalton Trans.* ***2018****, 47, 1159–117*
20	$[Cd_2Na(L_{15})(BDC)_{2.5}].9H_20$	DMF	1.67×10^4	162ppb	*J. Mater. Chem. A* ***2017****, 5, 15797–15807*
21	$[Zn_3(L_{16})_2(bipy)(\mu^3\text{-}OH)_2].3H_2O$	DMF	2.3×10^4	NA	*Sensors and Actuators B* ***2018****, 257, 207–213*
22	$[Tb_2(TDC)_3(CH_3OH)_2.(CH_3OH)]$	Methanol	9.52×10^3	NA	*RSC Adv.* ***2016****, 6, 91741–91747*
23	$Eu_2(MFDA)_2(HCOO)_2(H_2O)_6$	DMF	1.58×10^3	0.3μM	*Dalton Trans. 2013, 42,*

					12403-12409
24	**11a**	Water	1.91×10^4	0.219 µM /166 pbb	***This Work***

L_1 = 9,9-dimethylfluorene-2,7-dicarboxylic acid, L_2=4′-(4-carboxyphenyl)-2,2′: 6′,2″-terpyridine), L_3 = 4,4′-oxidibenzoic acid, L_4 = 3,5-bis(5-(pyridin-4-yl)thiophen-2-yl)pyridine, L_5= 3-(1H-pyrazol-3-yl) benzoic acid), L_6= 4,4′,4″-s-triazine-1,3,5-triyltri-m-aminobenzoate, L_7= 4,4′-(2,5-bis(methylthio)-1,4-phenylene)dipyridine, L_8 = 5,8-di(1*H*-imidazol-1-yl)quinoxaline, L_9 =5-hydroxyisophthalic acid, L_{10} = 2,4,6-tris[1-(3-carboxylphenoxy) ylmethyl]mesitylene, L_{11}=2,8,14,20-tetra-ethyl-6,12,18,24-tetra-methoxy-4,10,16,22-tetra-carboxy-methoxy-calix[4]arene, (H3TPT)= p-terphenyl-3,4″,5-tricarboxylic acid

Table S7. A comparison of quenching constants and corresponding LODs for various luminescent MOFs used for detection of $Cr_2O_7^{2-}/CrO_4^{2-}$

Entry	Material	Analyte	Solution	K_{sv} (M^{-1})	Detection Limit (M)	Reference
1	$[Zn(L_1)(L_2)]_n$	$Cr_2O_7^{2-}/CrO_4^{2-}$	H_2O	$1.37\times10^3/1.00\times10^3$	$1.202\times10^{-5}/1.833\times10^{-5}$	*Inorg. Chem.* **2017**, *56*, 2627-2638
	$[Cd(L_1)(L_2)]_n$	$Cr_2O_7^{2-}/CrO_4^{2-}$	H_2O	$2.91\times10^3/1.30\times10^3$	$2.26\times10^{-6}/2.52\times10^{-6}$	
2	$[Zn_2(L_3)(L_4)_2]\cdot4H_2O$	$Cr_2O_7^{2-}/CrO_4^{2-}$	DMF	$7.59\times10^3/4.45\times10^3$	$3.9\times10^{-6}/4.8\times10^{-6}$	*J. Mater. Chem. A.* **2016**, *4*, 15494-15500
3	Eu^{3+}@MIL-121	$Cr_2O_7^{2-}$		4.34×10^3	0.054 µM	*New J. Chem.* **2016**, *40*, 610 4654-4661
3	$[Eu(L_5)_2(NO_3)]\cdot H_2O$	$Cr_2O_7^{2-}$	ethanol		2.2×10^{-5}	*Inorg. Chem.* **2016**, *55*, 3952-3959
4	$\{[Cd(L_6)(L_7)]\cdot2H_2O\}n$	$Cr_2O_7^{2-}$	H_2O	6.4×10^3	3.76×10^{-5}	*Cryst. Growth Des.* **2017**, *17*, 67-72
	$\{[Cd(L_6)(L_8)(H_2O)]\cdot0.5H_2O\}_n$	$Cr_2O_7^{2-}$	H_2O	4.97×10^3	4.86×10^{-5}	
5	$[Eu_2(L_9)_4\cdot CO_3\cdot4H_2O]\cdot$DMF$\cdot$ solvent	$Cr_2O_7^{2-}/CrO_4^{2-}$	H_2O	$1.04\times10^4/4.85\times10^3$	$3.64\times10^{-6}/1.70\times10^{-6}$	*Inorg. Chem.* **2017**, *56*, 4197-4205
6	$[Zn(btz)]_n$	$Cr_2O_7^{2-}/CrO_4^{2-}$	H_2O	$4.23\times10^3/3.19\times10^3$	2/10	*CrystEngComm* **2016**, *18*, 4445-4451
	$[Zn_2(ttz)H_2O]_n$	$Cr_2O_7^{2-}/CrO_4^{2-}$		$2.19\times10^3/2.35\times10^3$	20/20	
7	$[Cd_6(L_{10})_2(bib)_2(DMA)_4]$	$Cr_2O_7^{2-}/CrO_4^{2-}$	H_2O			*Chem. Eur. J.* **2015**, *21*, 11475–11482
	$[Cd_3(L_{10})(tib)(DMF)_2]$	$Cr_2O_7^{2-}/CrO_4^{2-}$				
**8**	NU-1000	$Cr_2O_7^{2-}$	H_2O	1.33×10^4	1.8 µM	*Inorg. Chem.* **2017,** *56*, 14178−14188
9	$[Ni_2(\mu_2\text{-}OH)(azdc)(tpim)](NO_3)\cdot6DMA\cdot6MeOH$	$Cr_2O_7^{2-}/CrO_4^{2}$	H_2O	7.90×10^3/ 1.31×10^4	0.9 / 0.29 ppm	*ACS Appl. Mater. Interfaces* **2019**, *11*, *43*, 40134-40150
10	$[Y(BTC)(H_2O)_6]_n$:0.1Eu	$Cr_2O_7^{2-}/CrO_4^{2-}$		$1.18 \times 10^3/4.52 \times 10^3$	0.03/0.04µM	*Microporous Mesoporous*

						Mater. **2015**, *217*, 196-202
**11**	**`11a**	**$Cr_2O_7^{2-}/CrO_4^{2-}$**	**H_2O**	**2.18 × 10^4/1.46 × 10^4**	0.150 µM /0.236µM<br><br>114 ppb /179 ppb	**This work**

L_1=isophthalic acid (H_2IPA) and L_2=3-pyridylcarboxaldehyde nicotinoylhydrazone L_3(TPOM) = tetrakis(4-pyridyloxymethylene)methane L_4(NH_2–BDC) = 2-aminoterephthalic acid), L_5(H_2pzbc) = 3-(1H-pyrazol-3-yl) benzoic acid) L_6 = 4,4′-(2,5-bis(methylthio)-1,4-phenylene)dipyridine, L_7 = 4,4′-biphenyldicarboxylic acid, L_8 = 4,4′-sulfonyldibenzoic acid, L_9 (Htpbpc) = 4′-[4,2′;6′,4″]-terpyridin-4′-yl-biphenyl-4-carboxylic acid, L_{10}= hexa[4-(carboxyphenyl)oxamethyl]-3-oxapentane acid

Reference:

[1]Nandi, S., Haldar, S., Chakraborty, D., & Vaidhyanathan, R. (2017). Strategically designed azolyl-carboxylate MOFs for potential humid CO_2 capture. *Journal of Materials Chemistry A*, *5*(2), 535-543.
